# Hydrothermally tailored high-basalt geopolymer for strength development and indigo carmine removal

**DOI:** 10.1039/d6ra07821d

**Published:** 2026-10-16

**Authors:** Musa A. Said, Alaa Mohsen, Mostafa. A. Sayed, Fatma. M. Helmy, Mai M. Khalaf, Kawther Al-Amer, Hany M. Abd El-Lateef, Saad Shaaban, Nagih M. Shaalan, M. Ramadan

**Affiliations:** a Department of Chemistry, Faculty of Science, Islamic University of Madinah Madinah 42351 Saudi Arabia; b Faculty of Engineering, Ain Shams University Cairo Egypt; c Department of Chemistry, Faculty of Science, Ain Shams University Cairo 11566 Egypt m.ramadan@sci.asu.edu.eg; d Department of Chemistry, College of Science, King Faisal University Al-Ahsa 31982 Saudi Arabia mmkali@kfu.edu.sa; e Department of Physics, College of Science, King Faisal University Al-Ahsa 31982 Saudi Arabia

## Abstract

The development of sustainable multifunctional construction materials capable of simultaneously providing structural performance and environmental remediation has attracted considerable research interest. In this study, a low-carbon alkali-activated geopolymer was synthesized using an equal proportion of ground granulated blast-furnace slag (GGBFS) and Egyptian natural basalt as aluminosilicate precursors. The influence of NaOH concentration (5 and 10 wt%) and curing regime (ambient curing and hydrothermal treatment at 150 °C for 3–24 h) on the fresh, mechanical, phase, morphological, and textural characteristics of the developed composites was systematically investigated. Hydrothermal curing markedly enhanced geopolymerization, promoted pore refinement, and induced the formation of crystalline calcium aluminosilicate hydrate and zeolitic phases, resulting in a maximum compressive strength of 53.3 MPa for the Geo-BT5/H composite after 12 h of autoclaving. Owing to its superior mechanical integrity, well-developed meso-porosity, and low production cost, Geo-BT5/H was selected as an adsorbent for Indigo Carmine (IC) removal from aqueous solutions. Adsorption was strongly pH-dependent, exhibiting maximum performance under acidic conditions (pH ≈ 2), while adsorption capacity increased with both initial dye concentration and temperature. The BET analysis confirmed the transformation of the pore structure from a Type III to a Type IV/H3 mesoporous network with a specific surface area of 30.91 m^2^ g^−1^, which increased to 39.7 m^2^ g^−1^ following acid activation. Thermodynamic analysis demonstrated that IC adsorption is an endothermic, entropy-driven, and spontaneous process at elevated temperatures. Nonlinear equilibrium analysis showed that the Redlich–Peterson model provided the best fit to the experimental data, while kinetic modeling indicated that IC adsorption proceeds through a multistage process involving surface adsorption and intraparticle diffusion. Interference studies demonstrated the influence of coexisting species on IC removal, providing additional insight into the adsorption behavior of Geo-BT5/H under more complex aqueous conditions. Furthermore, the geopolymer maintained excellent structural stability and adsorption performance after repeated adsorption–desorption cycles, as confirmed by FTIR and XRD analyses. These findings demonstrate that hydrothermally cured slag-basalt geopolymers represent promising multifunctional materials that integrate high mechanical performance with efficient and reusable dye adsorption for sustainable wastewater treatment applications.

## Abstract 2

A hydrothermally tailored slag-basalt geopolymer with enhanced meso-porosity and zeolitic phases exhibits high strength and efficient indigo carmine adsorption for sustainable wastewater treatment.

## Graphical abstract

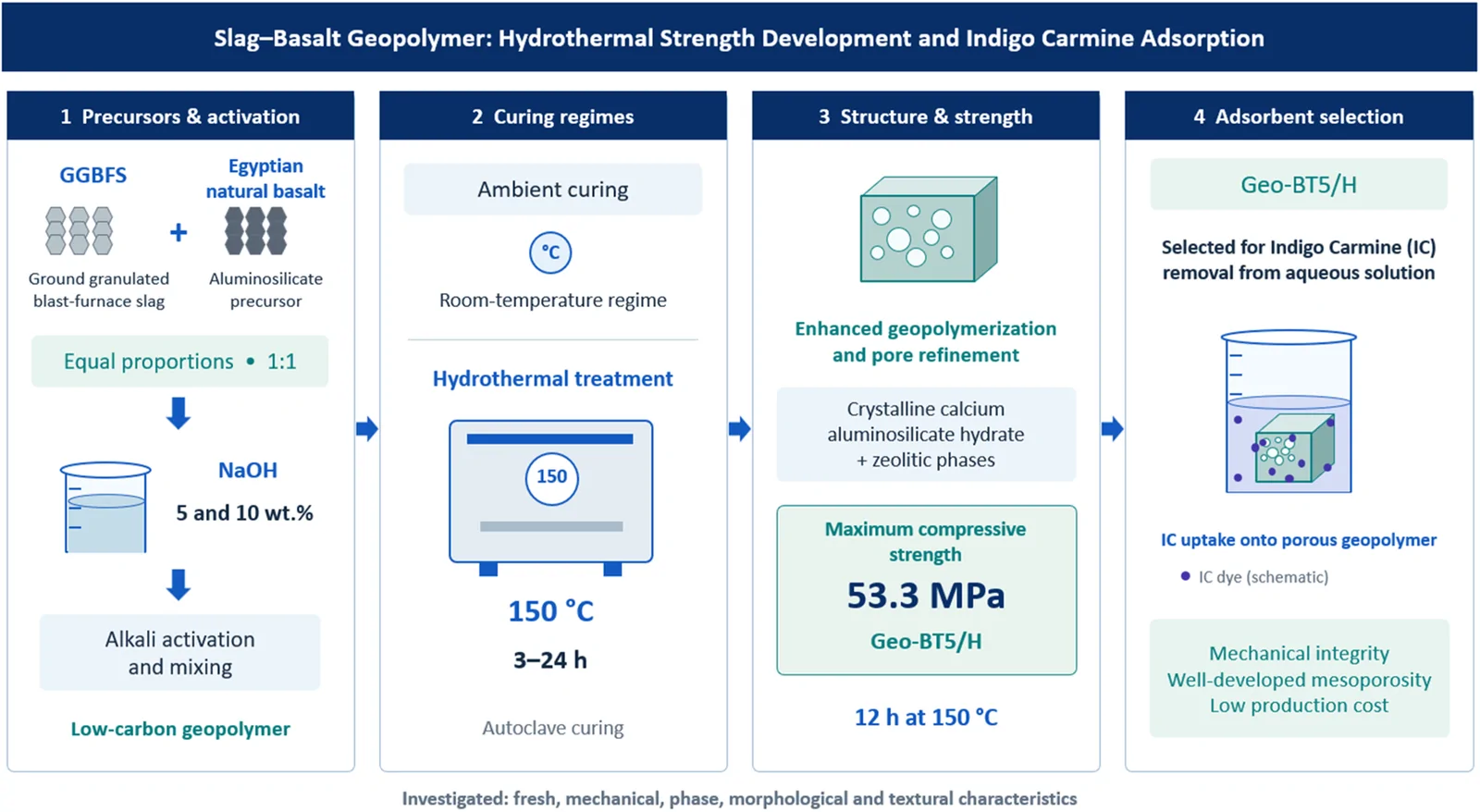

## 1 Introduction

The continuous expansion of the global construction sector heavily relies on ordinary Portland cement (OPC), a traditional binder whose production is notoriously energy-intensive and responsible for approximately 7–8% of anthropogenic CO_2_ emissions worldwide.^1,2^ To mitigate these environmental burdens and promote circular economy paradigms, alkali-activated materials (AAMs) and geopolymers have emerged as highly sustainable and low-carbon alternatives. They synthesized *via* the alkaline activation of solid aluminosilicate precursors, these materials offer satisfactory mechanical properties, enhanced durability, and significant reductions in greenhouse gas emissions compared to OPC.^3,4^

Aluminosilicate frameworks may be sourced from a diverse array of industrial residues, such as fly ash,^5^ silica fume,^6^ glass waste,^7^ slag,^8^ and various forms of sludge,^9^ in addition to being obtained from naturally abundant materials including basalt^10^ and clay deposits.^11^ Ground granulated blast-furnace slag (GGBS) is widely utilized due to its latent hydraulic reactivity and high calcium content. Alkali-activated slag predominantly forms highly cohesive calcium silicate hydrates (CSHs) and calcium aluminosilicate hydrates (CASHs), which provide exceptional early mechanical strength and dense microstructures.^12,13^ Nevertheless, the practical application of 100% slag-based binders is often hindered by several key limitations. Chief among these is the limited availability of reactive (amorphous) ground granulated blast furnace slag (GGBFS) in Egypt, which stems from the minimal reliance on blast furnace steelmaking relative to electric arc furnace technology. In addition, such systems are characterized by excessively rapid setting and pronounced autogenous shrinkage, further restricting their practical applicability.^14^ To address these drawbacks, recent research has focused on blending slag with natural, less reactive aluminosilicates. Basalt, a widespread igneous volcanic rock rich in silica, alumina, iron, and magnesium, has garnered considerable attention as a supplementary solid precursor.^15^

Recent studies have demonstrated that combining slag with basalt powder synergistically enhances the matrix, provided that the replacement level is optimized. For instance, Rashad *et al.*^16^ reported that incorporating Egyptian natural basalt powder into alkali-activated slag (AAS) systems at moderate levels (up to 20 wt%) not only densified the microstructure but also prolonged the initial and final setting times and mitigating the rapid workability loss typical of pure slag cements.

To establish a comprehensive baseline for integrating volcanic materials into sustainable binders, previous literature has documented the distinct numerical and physical impacts of basalt rock components on fresh and hardened milestones. Regarding fresh-state behaviors, altering the volcanic fraction systematically modifies the concrete fluid flow and rheological indicators. As monitored by Boğa and Şenol,^17^ utilizing waste basalt aggregates directly influences both the fresh and hardened performance profiles of high-strength self-compacting concrete configurations. Concurrently, variations in precursor dosing alter the hydration kinetics and initial solid-network formations. As verified by Venyite *et al.*,^18^ incorporating fine basalt powder into aluminosilicate-rich matrix systems systematically prolongs both initial and final setting times as the overall basalt amount rises.

In terms of mechanical performance and strength development, pure geopolymer pastes derived from 100% basalt powder typically achieve a lower structural efficiency and compressive strength compared to pure 100% slag-based configurations. However, combining these precursors reveals specific performance trade-offs under diverse service environments. For instance, Akturk and Ayhan^19^ monitored binary alkali-activated systems and confirmed that the final compressive strength and microstructural characterization are fundamentally dependent on the blending ratios of the basalt powder and slag matrices. Additionally, the precursor blending ratio governs the final durability under harsh environments; while alkali-activated slag mortars modified with a 20% waste basalt powder dosage experience a sharp decline in strength after exposure to elevated temperatures reaching up to 900 °C.^20^

The reaction kinetics, fresh properties, mechanical performance in addition to microstructure of alkali-activated these systems are fundamentally governed by the type and concentration of the alkaline activator. Sodium hydroxide (NaOH) is the most ubiquitous and low-cost activator, characterized by its high pH, which facilitates the aggressive dissolution of Si and Al species from the solid precursors to initiate the polycondensation process.^21^ The concentration of NaOH plays a critical role in dictating the fresh state properties of the pastes, particularly the initial and final setting times.

The literature suggests that alkaline activation parameters, particularly the composition and concentration of the activating solution, strongly influence the initial dissolution process and the formation of binding gels in geopolymer and alkali-activated materials.^22^ Previous investigations on fly ash- and GGBS-based systems have demonstrated that variations in activation conditions can modify setting time, early-age microstructure, and fresh-state workability.^13,23,24^ Generally, lower alkaline dosage is linked to slower early reaction kinetics and extended setting time, whereas higher dosage tends to accelerate early reactions and reduce the workable period, potentially promoting earlier strength development.^13,24^ Accordingly, quantifying setting behaviour under different alkaline activation levels is important for tailoring these binders to applications that demand an appropriate balance between workability and early-age performance.^13,22^

To further engineer microstructural architecture and enhance the performance of alkali-activated materials, advanced curing techniques such as hydrothermal treatment have been increasingly explored. Unlike conventional normal curing at ambient conditions, hydrothermal processing involves subjecting the materials to elevated temperatures and saturated pressures inside an autoclave. This highly reactive aqueous environment exponentially accelerates the dissolution of precursors, promotes rapid gel formation, and induces the partial recrystallization of amorphous phases into highly stable crystalline structures.^25,26^ For example, Boğa and Şenol *et al.*^17^ monitored geopolymer samples hydrothermally cured at 150 °C and observed the progressive evolution of typical amorphous humps into defined crystalline silicate and zeolitic phases over extended periods. Similarly, studies by Yang *et al.*^27^ on aluminosilicate-rich tailings demonstrated that hydrothermal activation effectively decomposes crystalline mineral phases and rapidly yields high early mechanical strength comparable to long-term ambient curing.

Indigo carmine (IC) is among the most widely used dyes in the textile industry, particularly for coloring denim. Beyond its industrial applications, IC demonstrates notable versatility in medical diagnostics and surgical procedures, including microsurgery, gynecological, and urological operations. However, studies have indicated that IC poses health risks to humans, potentially causing gastrointestinal disturbances, skin irritation, and hypotension.^28,29^

Geopolymers have emerged as promising materials for treating wastewater contaminated with industrial dyes, particularly cationic dyes, due to their distinctive surface characteristics, such as crosslinking capabilities, inherent negative charges, and porous microstructures. Among the various technologies employed for dye removal, adsorption stands out for its energy efficiency, cost-effectiveness, and scalability in industrial applications.^30^ The synthesis of functionalized geopolymer adsorbents derived from industrial byproducts has opened promising avenues for environmental remediation, particularly in the removal of synthetic dyes from textile wastewater. Previous investigations have systematically focused on evaluating the efficacy of ground granulated blast furnace slag (GGBFS)-based geopolymers for the adsorptive removal of Indigo Carmine (IC) dye from aqueous solutions. These studies demonstrated that the geopolymer's porous architecture, high surface area, and variable elemental precursor ratios significantly contribute to an outstanding dye removal efficiency under optimized dosage, contact time, and controlled pH environments.^31,32^ While these conventional systems successfully highlight the utility of pure high-calcium slag frameworks for water remediation,^33^ a critical research trajectory lies in exploring how blending slag with natural minerals alters these macro-porous properties. Consequently, the development of a binary precursor system presents an innovative approach to evaluate whether incorporating fine volcanic rock fractions can complement the primary slag matrix, thereby offering dual functionality in both high-strength construction and enhanced hazardous dye remediation.

Although previous studies have investigated alkali-activated GBFS/basalt blends with varying slag-to-basalt ratios, important knowledge gaps remain regarding the behavior of high-basalt-content systems. In particular, the combined effects of alkali activator concentration and hydrothermal curing on the reaction development, phase evolution, pore structure, and mechanical performance of a fixed 1 : 1 slag-basalt composition (50 wt% basalt) have not been systematically examined. Moreover, it remains unclear whether hydrothermal treatment can compensate for the lower reactivity of basalt and promote the formation of a dense and mechanically robust binder at such a high replacement level. While dye removal by alkali-activated materials has been reported, the adsorption behavior of high-basalt-content slag-basalt binders and the relationship between hydrothermal curing-induced microstructural evolution, meso-porosity, mechanical properties, and dye adsorption performance have not been comprehensively established.

To address these gaps, this study develops a high-basalt-content alkali-activated slag-basalt composite containing 50 wt% natural basalt, a composition that is particularly attractive in Egypt due to the abundance, local availability, and low cost of basalt. The study systematically evaluates the effects of NaOH concentration (5 and 10 wt%) and conventional *versus* hydrothermal curing at 150 °C for 3–24 h on the reaction mechanisms, pore structure evolution, microstructure, and mechanical properties of the 1 : 1 slag-basalt system. In addition, it investigates whether hydrothermal curing can offset the reduced slag content and enhance binder densification and strength development. A further novelty is the establishment of direct correlations between hydrothermally induced microstructural and meso-porous characteristics, compressive strength, and indigo carmine adsorption capacity. Therefore, rather than treating hydrothermal curing, porous phase development, mechanical performance, and dye adsorption as independent aspects, this work integrates them into a unified structure–property-function framework, resulting in a low-cost, low-carbon, dual-functional geopolymer with potential applications in both construction and wastewater treatment.

## 2 Material and experimental program

### 2.1 Materials

Two aluminosilicate source materials were employed in this study: highly reactive amorphous ground granulated blast furnace slag (GGBFS) and Egyptian natural basalt (BT). The GGBFS was obtained from Lafarge Company in Ain El-Sokhna, Egypt, while the basalt was supplied by Sinai Cement Company in El-Arish, Egypt. Commercial-grade sodium hydroxide (NaOH), used as the alkaline activator, was purchased from Alpha-Aromatic Company in Cairo, Egypt. Indigo Carmine (IC) dye was acquired from LOBA, India.

Table 1 shows the chemical composition of GGBFS and BT. XRF technique (Termo-Scientific; ARL 9900-model) indicates that GGBFS is a calcium-rich aluminosilicate with high CaO, SiO_2_, and Al_2_O_3_ contents, indicating strong reactivity. BT has similar SiO_2_ and Al_2_O_3_ levels, along with notable CaO and Fe_2_O_3_. These outcomes confirm that BT is a suitable aluminosilicate precursor for geopolymer applications. Additionally, X-ray diffraction (XPert-2000; Philips) reveals the phases composition and crystallinity level of BT and GGBFS. As shown in Fig. 1, GGBFS exhibits a broad hump, indicating its semi-crystalline nature (44.32%), with minor peaks corresponding to gehlenite, akermanite, and calcite. In contrast, BT shows a high degree of crystallinity (82.8%), mainly composed of anorthite, labradorite, augite, and magnetite. The degree of crystallinity for both raw materials was calculated from the X-ray diffraction data using the Degree of Crystallinity (DOC) analysis function in Match! software (Version 3.15).

**Table 1:** XRF-analysis to identify oxide compositions of raw materials (wt%)

Material	SiO_2_	Al_2_O_3_	Fe_2_O_3_	CaO	MgO	TiO_2_	SO_3_	Na_2_O	K_2_O	Cl	LOI
GGBFS	50.01	14.11	0.78	24.50	6.32	—	1.78	0.52	0.86	0.61	0.51
BT	48.38	14.30	16.40	11.80	2.85	2.03	0.23	1.58	1.18	0.11	0.31

**Fig. 1:**
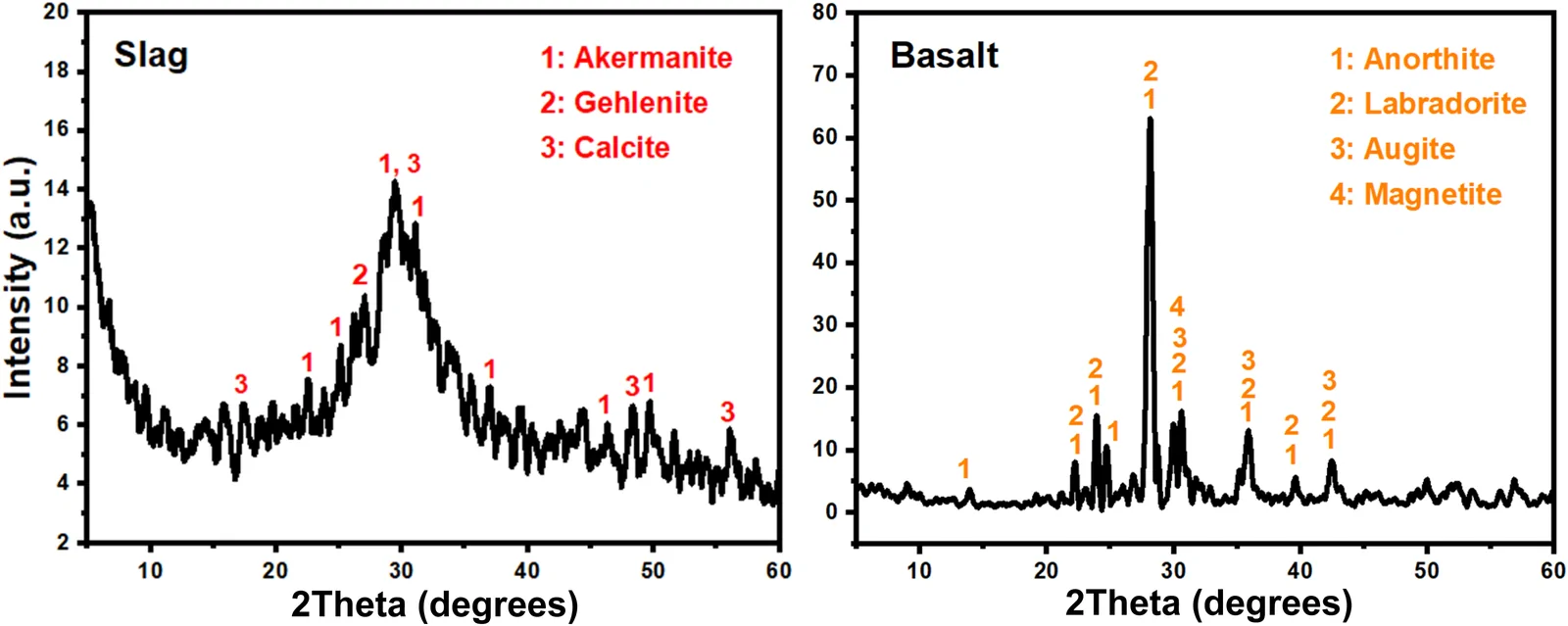
XRD-patterns of raw materials.

Particle size distribution analyzer (NICOMP; Nano-N-3000) shows a clear difference between the two precursors (Fig. 2). Generally, PSD significantly influences the performance of geopolymer composites (Geo-BT), affecting physico-mechanical, and removal efficacy. The dominant particle sizes are 11.67 µm for GGBFS and 2.96 µm for BT, indicating the finer nature of BT.

**Fig. 2:**
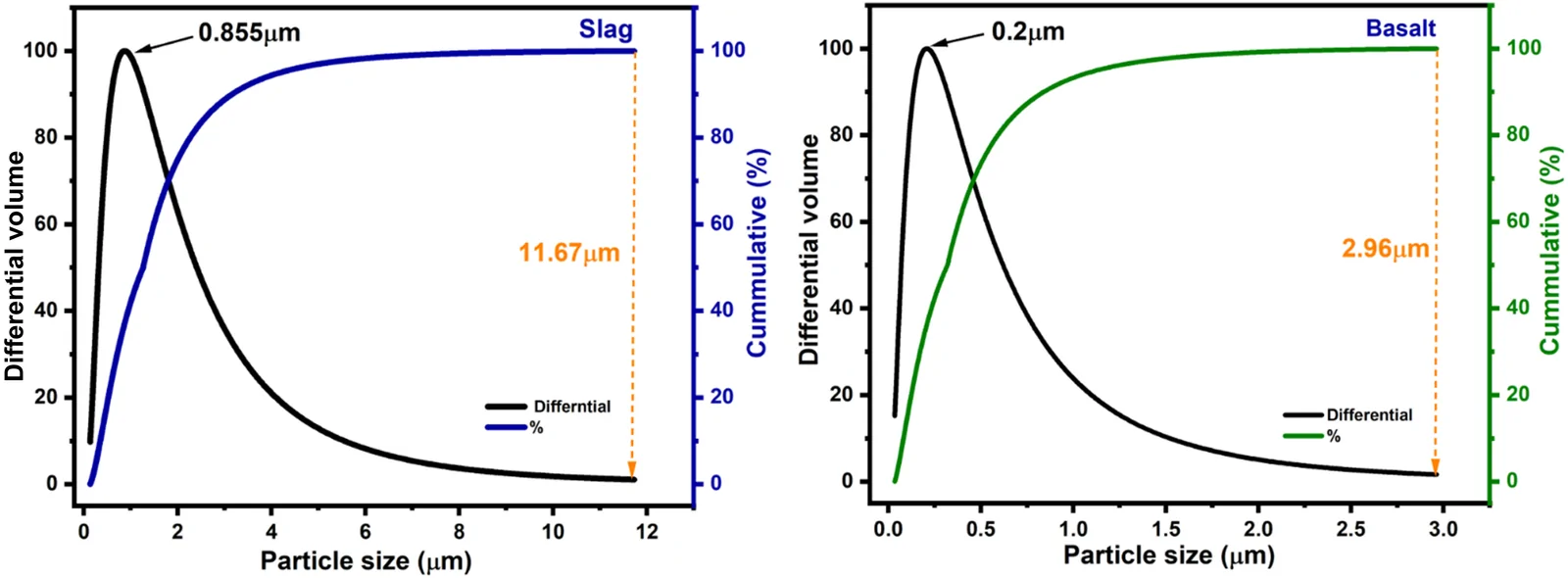
Particle size distribution curves of raw materials.

### 2.2 Experimental program

#### 2.2.1 Casting of geopolymer pastes

First, two separate dry blends were produced by mixing GGBFS (400 g) and BT (400 g) in a 1 : 1 ratio using a porcelain ball mill for 120 minutes. Then, the first blend was activated with a 5 wt% NaOH solution (40 g NaOH dissolved in 240 ml of water), while the second blend was activated with a 10 wt% NaOH solution (80 g NaOH dissolved in 240 ml of water). The prepared alkaline solutions were allowed to cool to room temperature before combined with the aluminosilicate precursors. Based on the previous literature,^7–9,34^ the mixing water for each composition was maintained at 30% (W/B = 0.3) to ensure suitable flowability. An ELE automatic mixer was employed for 180 seconds to blend the dry binders with the alkaline activator solution, producing workable pastes (Geo-BT5 and Geo-BT10) ready for casting into cubic stainless-steel molds (5 × 5 × 5 cm).^35^ OPC (control) pastes^36^ were prepared with the same W/B ratio without alkaline activator. A vibrating table was then used to eliminate entrapped air and achieve dense, compact pastes after hardening. The cast specimens were cured for 24 hours at ambient temperature under high relative humidity. After this initial hardening period, the alkali-activated pastes were divided into two groups: the first group was cured under high humidity conditions for 2 and 28 days, while the second group was subjected to autoclave curing at 150 °C for 3, 6, 12, and 24 hours. These curing regimes were selected based on previous studies reported in the literature.^9,34,37–39^Table 2 indicates mix-design and codes of fabricated pastes while Fig. 3 displays a schematic diagram representing preparation of geopolymeric pastes.

**Table 2:** Mix design and codes of fabricated pastes (wt%)

Composite	OPC (%)	GGBFS (%)	BT (%)	NaOH (%)	W/B	Curing regime
OPC	100	—	—	—	0.3	Curing under water at 30 °C for 28 days
Geo-BT5/N	—	50	50	5	0.3	Normal treatment in high humidity at 30 °C for 28 days
Geo-BT10/N	—	50	50	10	0.3	Normal treatment in high humidity at 30 °C for 28 days
Geo-BT5/H	—	50	50	5	0.3	Hydrothermal curing at 150 °C for 3, 6, 12, 24 h
Geo-BT10/H	—	50	50	10	0.3	Hydrothermal curing at 150 °C for 3, 6, 12, 24 h

**Fig. 3:**
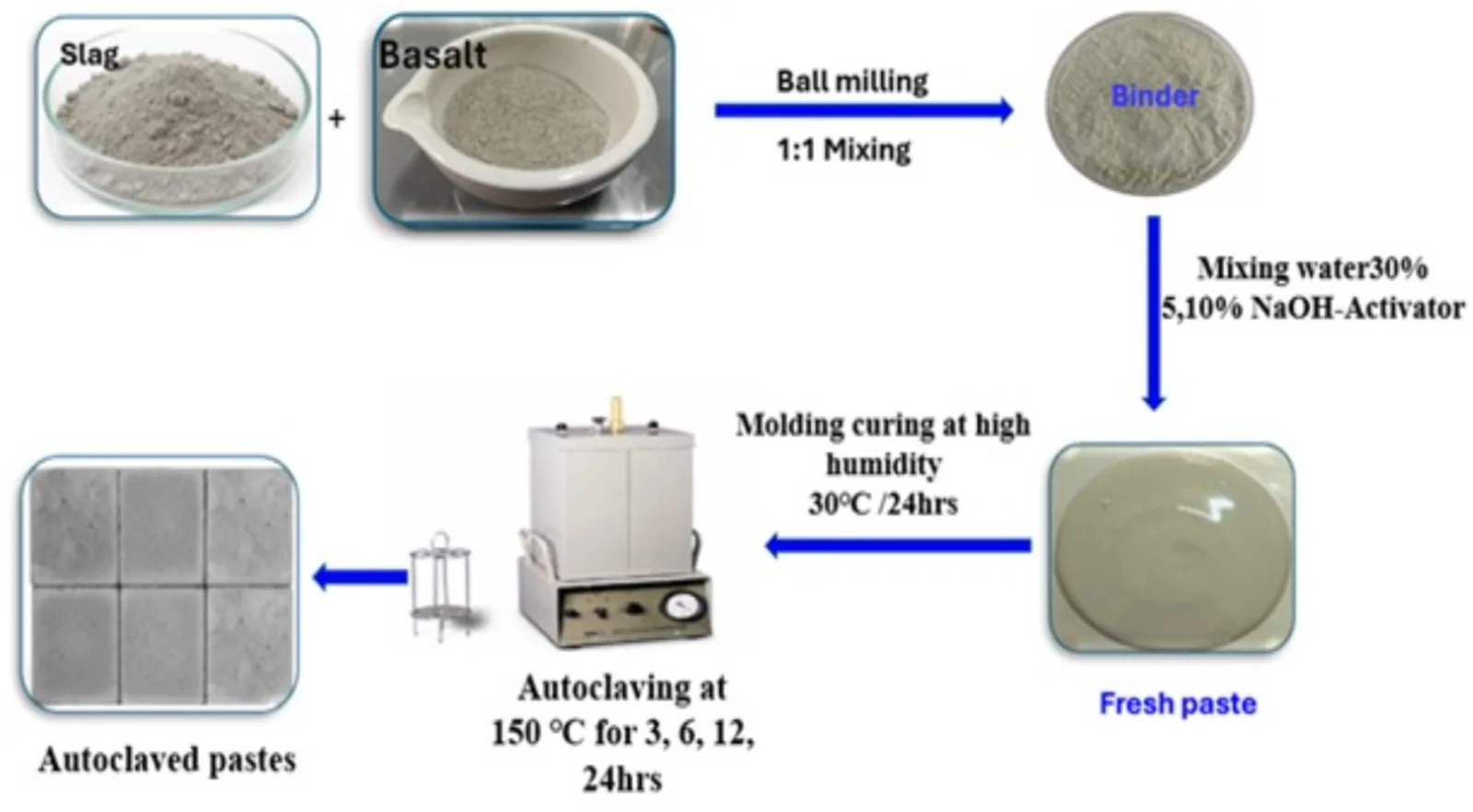
Flowchart illustrating the preparation process of autoclaved geopolymer pastes.

#### 2.2.2 Physicomechanical testing and characterization

The flowability of the fresh pastes (OPC, Geo-BT5, and Geo-BT10) was assessed using a modified mini-slump test, where the spread diameter was used as an indicator of flow characteristics. The measured spread diameter was used to calculate the corresponding spread area, with a larger spread area indicating greater workability of the paste.^36^ The initial and final setting times were determined using a Vicat apparatus in accordance with ASTM C191.^40^ The compressive strength of the hardened pastes, was evaluated using four cube specimens for each composite under the different curing regimes, following ASTM C109M (2013).^41^ Testing was performed using a Ton-Industrie compression testing machine with a maximum load capacity of 60 tons. To investigate the physicochemical characteristics of the synthesized geopolymeric pastes, selected samples were analyzed *via* XRD, TGA/DTGA, FTIR, BET/BJH and SEM/EDX techniques to characterize the phase assemblage, thermal behavior, functional groups, pore structure, and morphology of the formed binding hydrates.

#### 2.2.3 Dye adsorption experiments

From both economic and performance perspectives, the Geo-BT5 specimen autoclaved at 150 °C for 12 h (Geo-BT5/H) was selected as the optimal adsorbent material due to its favorable balance of low production cost, high mechanical strength, and well-developed textural properties, which make it highly suitable for adsorption applications. Following compressive strength determination, the crushed samples were finely pulverized and sieved to below 200 mesh to ensure adequate particle fineness for further adsorption studies.

##### 2.2.3.1 Effect of pH

The effect of solution pH on the adsorption of indigo carmine (IC) was evaluated by contacting 0.40 g of sorbent powder with 50.0 mL of IC solution (initial concentration 20.0 mg L^−1^) at ambient temperature. Solution pH was adjusted over the range 2.0–12.0 using 0.10 M NaOH and 0.10 M HCl and monitored with a calibrated pH meter. Suspensions were agitated at 250 rpm for 120 min before sampling, and residual dye concentrations were determined by a double-beam UV-vis spectrophotometer. Additionally, hydrothermally cured geopolymer specimens (Geo-BT5/H) were subjected to acid activation to enhance surface properties and adsorption performance under acidic conditions. Powdered geopolymer samples were treated with 1.0 M HCl at a solid-to-liquid ratio of 1 : 10 (w/v) with continuous stirring at ambient temperature for 2 h or until the solution pH reached approximately 2.0. The acid-activated materials were then repeatedly rinsed with deionized water until neutral pH was achieved and dried at 80 °C for 12 h. The resulting activated sorbents were characterized by BET/BJH surface area and porosity analysis and employed in subsequent dye adsorption experiments.

##### 2.2.3.2 Surface analysis

The point of zero charge (pHPZC) of the synthesized adsorbent was determined following the procedure of Dahri *et al.*^42^ Experiments were carried out in 100 mL Erlenmeyer flasks, each containing 25.0 mL of 0.10 M KNO_3_ and 0.05 g of adsorbent powder. The initial pH of the KNO_3_ suspensions was adjusted over the range 2.0–12.0 using 0.10 M HCl and 0.10 M NaOH. Suspensions were agitated vigorously at 25 °C for 24 h, after which the final pH was recorded. The pHPZC was obtained by plotting ΔpH (final pH − initial pH) *versus* initial pH and identifying the pH at which ΔpH = 0.

##### 2.2.3.3 Equilibrium studies

Batch adsorption experiments were performed by contacting 0.40 g of sorbent with 50.0 mL of dye solution at initial concentrations of 10–100 mg L^−1^. Suspensions were agitated at 250 rpm for 120 min at ambient temperature, after which residual dye concentrations were determined by UV-vis spectrophotometry. Each experiment was carried out in triplicate (*n* = 3); reported values are arithmetic means and error bars in the figures represent the corresponding standard deviations. Removal efficiency (%) of the adsorbents was calculated using:1
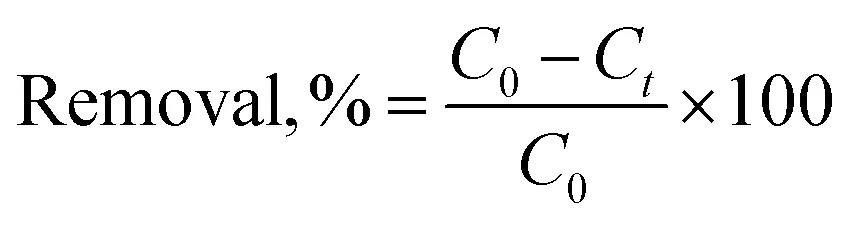
where *C*_0_ and *C*_*t*_ (mg L^−1^) are the initial and proceeding concentrations of the adsorbate (IC), respectively. The adsorption capacity, *Q*_e_ (mg g^−1^), was calculated at equilibrium *via*eqn (2):2
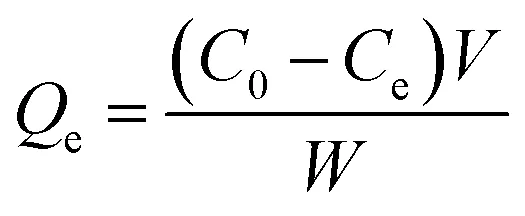
where *C*_0_ (mg L^−1^) and *C*_e_ (mg L^−1^) represent the concentrations of the IC dye at the beginning and at equilibrium, respectively. The volume of the solution is *V* (L), and the mass of the dry sorbent (*W*) used is expressed in grams.

##### 2.2.3.4 Thermodynamic studies

To evaluate the influence of temperature on the characteristics of the investigated adsorption processes, the thermodynamic parameters—Gibbs free energy (Δ*G*°), standard enthalpy change (Δ*H*°), and standard entropy change (Δ*S*°)—were calculated using the following Van't Hoff equations:^43^3
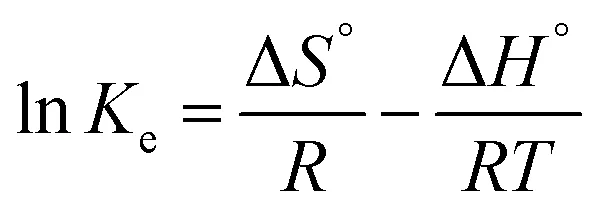

4 Δ*G*° = Δ*H*° − *T*Δ*S*°

5
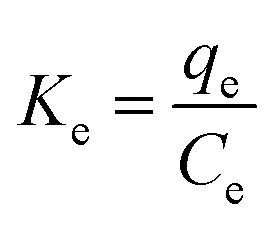
where *K*_e_ is the thermodynamic equilibrium constant, *q*_e_ is the amount of adsorbate adsorbed from the solution at equilibrium (mg L^−1^), *C*_e_ is the equilibrium concentration of adsorbate in the solution (mg L^−1^), *R* is the universal gas constant (8.314 J mol^−1^ K^−1^), and *T* is the temperature in (K).

##### 2.2.3.5 Adsorption isotherm models

The equilibrium data obtained for IC dye onto Geo-BT5/H were modeled with the Langmuir (eqn (6)), Freundlich (eqn (7)), Temkin (eqn (8)), Redlich–Peterson (eqn (9)), and Hill (eqn (10)) isotherm models. The nonlinear forms of the five isotherms are:6
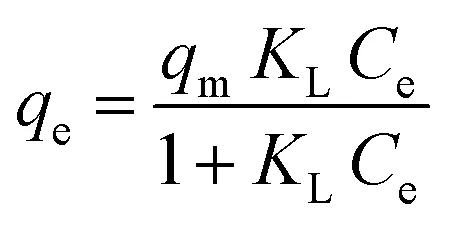

7 *q*_e_ = *K*_F_*C*_e_^1/*n*^

8 *q*_e_ = *B*_T_ln *K*_T_*C*_e_

9
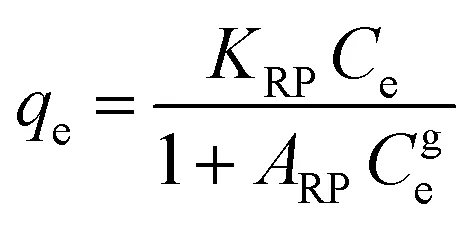
10
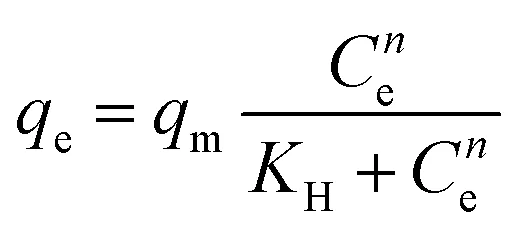
where *C*_e_ (mg L^−1^) and *q*_e_ (mg g^−1^) are the equilibrium concentration, and the amount of IC dye adsorbed at equilibrium, respectively. *K*_L_ (L mg^−1^) and *q*_m_ (mg g^−1^) are Langmuir constant, and the monolayer adsorption capacity, respectively. *K*_F_ and *n* are the Freundlich constants, which are indicators of adsorption capacity and adsorption intensity, respectively. Temkin constant (*B*_T_) is related to the heat of adsorption (kJ mol^−1^), *K*_T_ (L g^−1^) is the Temkin equilibrium binding constant. *K*_RP_ (L g^−1^) and *A*_RP_ (L mg^−1^) are the Redlich–Peterson constants; *g* is an exponent whose value must lie between 0 and 1. *K*_H_ (mg L^−1^) is the Hill constant that represents the concentration for which *q*_e_ is equal to 0.5 *q*_m_, and *n* (dimensionless) is the Hill coefficient of sigmoidicity which is related to the degree of cooperation between binding sites.

##### 2.2.3.6 Kinetics models

Lagergren's pseudo-first-order (eqn (11)), Ho and McKay's pseudo-second-order (eqn (12)), and the Elovich model (eqn (13)) adsorption kinetics models were used for fitting the kinetic adsorption data. The mechanism of adsorption onto Geo-BT5/H was investigated using Weber and Morris's intraparticle diffusion (eqn (14)):

11 *q*_*t*_ = *q*_e_(1 − e^−*K*_1_*t*^)

12
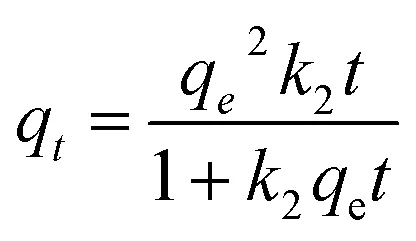
13
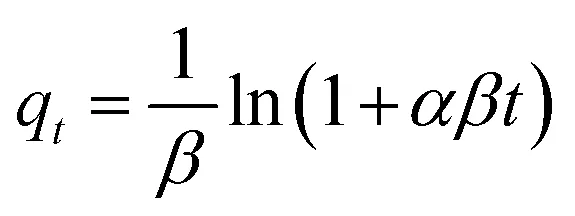

14 *q*_*t*_ = *k*_id_*t*^1/2^ + *C*_i_

where *q*_e_ (mg g^−1^) and *q*_*t*_ (mg g^−1^) are the amounts adsorbed at equilibrium and at time *t*, respectively. *k*_1_ (min^−1^) and *k*_2_ (g mg^−1^ min^−1^) are the pseudo-first order and pseudo-second order adsorption rate constants, respectively. *k*_id_ (mg g^−1^ min^−1/2^) is the intraparticle diffusion rate constant, and *C* is a constant related to the diffusion resistance. *α* (mg g^−1^ min) is the initial rate constant and *β* (g mg^−1^) is the desorption constant.

## 3 Results and discussions

### 3.1 Fresh properties

The fresh-state behavior of cementitious materials generally determines the ease of their casting, placement and finishing. It is dependent on the physical and chemical characteristics of the binder. Fig. 4a shows the initial/final-setting time (I/F-ST) of three systems: OPC as a reference paste and two geopolymeric materials composed of 50%GGBFS+50%BT activated with 5 and 10 wt% NaOH (coded: Geo-BT5 and Geo-BT10, respectively). The data shows that replacing OPC with geopolymeric materials (Geo-BT5 and Geo-BT10) markedly prolongs the I/F-ST. The recorded I/F-ST of OPC is 152/185 min, while for Geo-BT5 is 201/304 min and for Geo-BT10 is 237/334 min. It can be attributed the short I/F-ST of the OPC to the rapid hydration of its phases, such as tricalcium aluminate and tricalcium silicate, which participate in the formation of calcium-aluminate-hydrate (CAH), calcium-silicate-hydrate (CSH) and calcium hydroxide. These formed phases rapidly accumulated between the cement grains and then connected the solid skeleton.^44,45^ In contrast, the I/F-ST in the geopolymeric materials is controlled by the rate of dissolution of the aluminosilicate precursor, polycondensation and precipitation of calcium-alumino-silicate-hydrate (C-A-S-H) binding gel. These multi-stage reactions commonly need a long period, especially in the cementitious matrix containing partially inert or less reactive aluminosilicate precursors. This result is matched with Suwan and Fan.^46^ They reported that the I/F-ST of the OPC is shorter than that of fly-ash-based and geopolymer and they attributed this result to the low calcium content of the fly ash. Similarly, Buyondo *et al.*^47^ found the same observation in the rice-husk-ash-based geopolymer and they reported that the low reactivity and high silica content in the rice husk ash are the main reasons behind this behavior. Accordingly, the mineralogical nature of the starting precursors provides the principal explanation of this behavior. GGBFS is a highly reactive material under an alkaline medium, participating in the formation of reactive Ca/Si/Al species. On the contrary, the high-crystallinity/low-reactivity of BT refers to its low dissolution rate in the alkaline medium, in line with Abd Al-Aziz *et al.*^48^ and Venyite *et al.*^18^ Consequently, the BT can be considered as a slow-reacting mineral that delays the kinetics of setting. Interestingly, noticing that the balance between the GGBFS and BT in the developed matrix makes the I/F-ST in the acceptable range required for the binding materials in the construction application (between 45 and 375 min), according to ASTM C150/C150M-24.^49^ Regarding the impact of the concentration of NaOH on the setting time, as illustrated above, increasing the NaOH wt% causes elongation in the I/F-ST. At a low NaOH concentration, the dissolution of alumina and silica species from aluminosilicate precursors is low; however, the dissolution of Ca species is not interrupted. Accordingly, the bulk solution of the matrix becomes saturated with Ca^2+^ ions, causing fast formation of C-A-S-H, and then shortening the I/F-ST.^24,50,51^ On the other hand, increasing the concentration of NaOH improves the alumina and silica dissolution, making the solution saturated with them. This behavior hinders the dissolution of the Ca species, delaying the formation of the binding gel and elongating the I/F-ST.^52^ This observation and discussion were reported previously by Mohsen *et al.*^34^

**Fig. 4:**
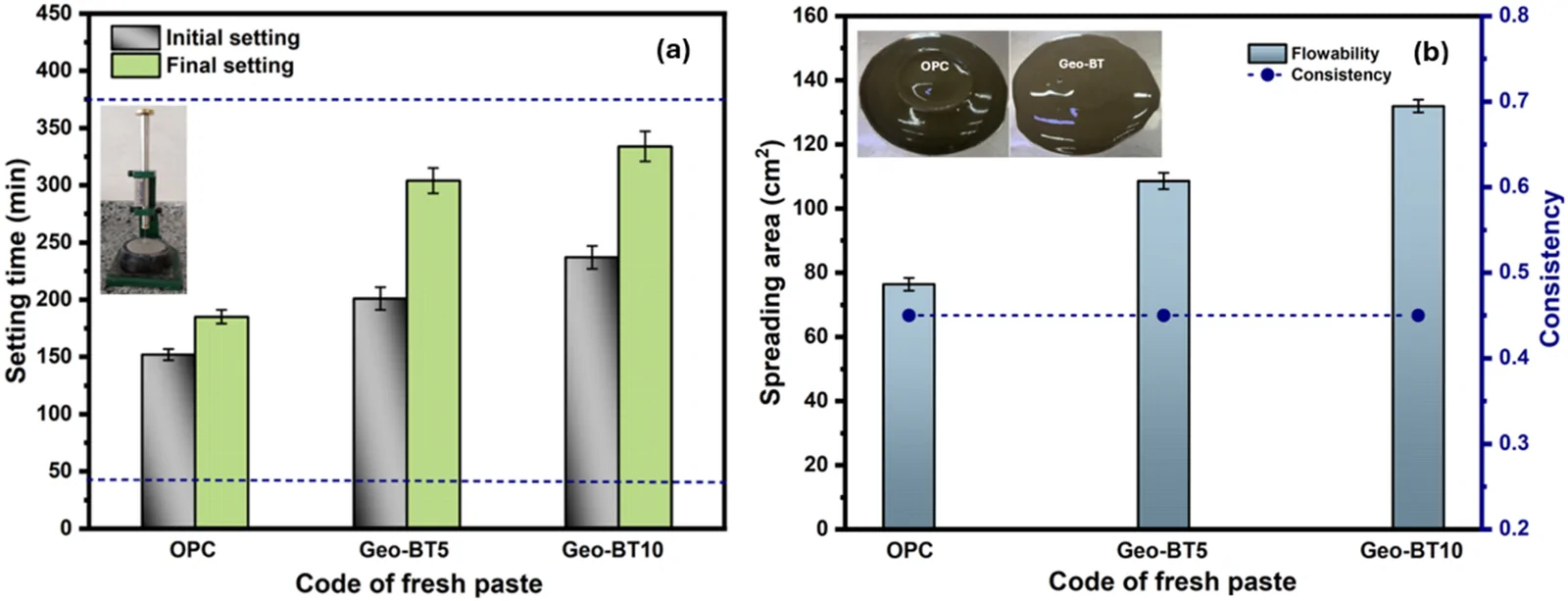
Fresh properties of the developed geopolymeric composites (a) setting time and (b) workability.

The workability of the developed composites is expressed by the spread area; an increase in the spread area indicates high workability.^53–55^ Compared with OPC, Geo-BT5 and Geo-BT10 exhibit an increase in the spread area by 42.1 and 72.6%, respectively, as represented in Fig. 4b. The low workability of OPC relative to the prepared geopolymeric composites may result from the rapid hydration of the OPC and the quick formation of early hydration products. Such products increase the interparticle cohesion and reduce the workability of fresh paste. In contrast, the prepared geopolymeric composites (Geo-BT5 and Geo-BT10) show an elongation in the setting time, maintaining a higher mobility of particles in the matrix. These data matched with Brandvold *et al.*^56^ when comparing the rheological properties of OPC with metakaolin-based geopolymer. Furthermore, owing to the higher crystallinity of the BT and its lower early chemical reactivity, it delays the formation of the binding gel responsible for the stiffness of the fresh paste. El-Didamony *et al.*^57^ found that as the amount of basalt in the blended cement increases, the water consistency decreases, referring to its role in increasing the workability. They attributed this behavior to the unhydraulic nature of BT in comparison to OPC. Regarding the higher workability of the Geo-BT10 compared with the Geo-BT5, it suggests that the high concentration of NaOH can enhance the dispersion of the particles. In addition to the elongation of the setting time with increasing the NaOH wt%, the highly alkaline environment can modify the surface charge of the aluminosilicate precursors. The existence of an additional amount of the OH^−^ creates an electrostatic repulsion force, reducing the particles' agglomeration and then enhancing workability, matching with Mohsen *et al.*^34^

### 3.2 Compressive strength

To determine the suitability of developed composites in the construction application, the compressive-strength of the prepared composites was measured as shown in Fig. 5. The results reveal a strong dependence of the compressive strength on the physico-chemical properties of the precursor, NaOH concentration and curing regime.^58^ Under normal curing conditions for 28-days (Fig. 5a), OPC has the highest strength values, reaching 42.5 MPa. Geo-BT5 and Geo-BT10 have compressive-strengths lower than OPC by 16.0 and 32.3%, respectively. This reduction in the strength values may result from the slow geopolymerization reaction kinetics of the GGBFS/BT matrix compared with OPC hydration. Once the particles of the OPC were hydrated, they formed C–S–H, densifying the microstructure and enhancing the mechanical performance.^59^ On the other hand, geopolymeric materials depend on the rate of dissolution of the precursors (GGBFS and BT), followed by the polycondensation and precipitation of the binding gel, which governs strength development.^60,61^ Partially replacing GGBFS with 50 wt% BT hinders the geopolymerization process and the formation of strength-giving-phases (C-A-S-H). The highly-crystalline/less-reactive BT that contains stable phases, such as anorthite, labradorite, augite and magnetite, is the main reason behind this behavior.^48^ Also, the data represents that increasing the concentration of NaOH in the GGBFS/BT-based geopolymer is combined with a reduction in the strength, reaching 19.3% when NaOH wt% increased from 5 to 10 wt%. Despite increasing NaOH wt% is commonly expected to improve aluminosilicate precursor dissolution, the obtained results indicate that excessive alkalinity did not improve the final mechanical performance. These results align with Ramadan *et al.*^11^ They showed that increasing the concentration of NaOH from 6 wt% to 8 wt% enhances the compressive strength of GGBFS/bentonite-based geopolymer. However, beyond this point, at 10 wt%, the compressive strength begins to decrease. Similarly, alkali activation of GGBFS/glass-waste-based geopolymer with 5 wt% NaOH yields a compressive strength higher than that of others activated with 10 wt% NaOH, as obtained by Mohsen *et al.*^34^ It can be attributed the reduction in the compressive-strength to (i) the rapid geopolymerization rate during the early stages, causing the accumulation of hydration products around unreacted GGBFS and BT particles, limiting their further dissolution;^62^ (ii) increasing the NaOH solution concentration hindered ion mobility, thereby delaying the geopolymerization process;^62^ and (iii) the excess Na^+^ ions create a screening (electrostatic shielding) of the silanol group (Si–OH) of aluminosilicate precursor particles, which prevents further dissolution.^63^

**Fig. 5:**
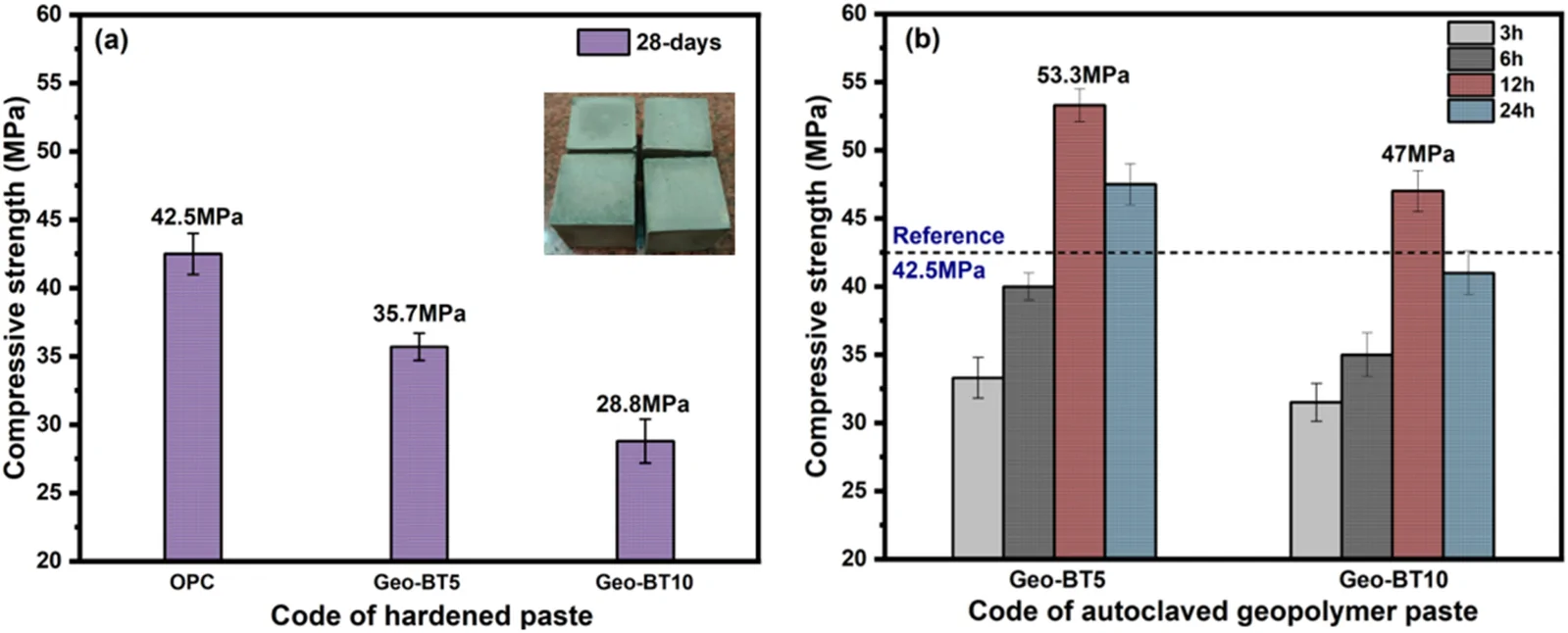
Strength of geopolymeric composites (a) 28-days normal curing and (b) hydrothermal curing.

Under hydrothermal curing conditions, the impact of curing of the developed composites at 150 °C for 3, 6, 12 and 24 h was evaluated to determine the optimum curing period, as graphically represented in Fig. 5b. Both Geo-BT5 and Geo-BT10 show a progressive enhancement in the compressive-strength from 3 to 12 h, followed by a decline after 24 h, concluding that 12 h is the optimum curing period. From 3 to 12 h, the compressive-strength increased by 60.1 and 49.2% for Geo-BT5 and Geo-BT10, respectively. Interestingly, noticing that the compressive-strength of Geo-BT5 and Geo-BT10 reaches 53.3 and 47.0 MPa, higher than normal curing by 49.3 and 63.2%, respectively. Under the combined effects of alkaline activation and a pressurized hydrothermal environment, the dissolution kinetics of Si- and Al-bearing species from the precursor phases are expected to increase, promoting further formation of geopolymeric reaction products and matrix densification.^9^ This causes the formation of additional strength-giving-phases integrated with a complex matrix from zeolitic phases characterized by superior hydraulic natures,^64^ as will be proved below by XRD and SEM. Moreover, hydrothermal curing results in redistributing/rearranging the pore structure, forming a denser matrix, as will be confirmed below by BET/BJH.^65^ On the contrary, increasing the curing period to 24 h slightly declines the compressive-strength due to increasing the internal stress, which causes the formation of microcracks, deteriorating the microstructure,^66^ in line with Sayed *et al.*^67^

### 3.3 Pore structure analysis

The pore structure of the synthesized geopolymeric pastes were analyzed using N_2_ adsorption–desorption isotherms and BJH-pore size distribution analyses (Fig. 6), while the textural parameters are summarized in Table 3. All investigated pastes were prepared from a slag-basalt precursor (1 : 1); however, the alkali activator concentration differed, with Geo-BT5 activated using 5 wt% NaOH and Geo-BT10 activated using 10 wt% NaOH. Therefore, the evolution of pore structure was influenced by both the curing regime and the activator concentration. The geopolymer specimens cured under ambient conditions for 28-days exhibited Type III adsorption isotherms,^5,59^ indicating weak N_2_-geopolymer interactions and the predominance of relatively large/macro-pores (Fig. 1–6). Among the ambient-cured geopolymer, Geo-BT5/28d presented a BET-surface area of 5.94 m^2^ g^−1^ and a total pore volume of 0.078 cm^3^ g^−1^, whereas Geo-BT10/28d showed lower values of 2.65 m^2^ g^−1^ and 0.043 cm^3^ g^−1^, respectively. The corresponding BJH pore size distributions (Fig. 1–6) revealed dominant pore diameters of 35.6 and 105.6 nm for Geo-BT5/28d (black curve) and Geo-BT10/28d (blue curve), respectively. The more open pore structure observed in Geo-BT10/28d, with a dominant pore diameter of 105.6 nm, indicates that a higher NaOH concentration adversely affected pore refinement during ambient curing. Although a higher alkali dosage enhances precursor dissolution, excessive hydroxyl ion concentration can destabilize the developing aluminosilicate network by promoting the disintegration of cross-linked gel structures and inhibiting the formation of a well-condensed three-dimensional framework.^62,68^ This phenomenon leads to the generation of macropores and a reduction in matrix compactness, which is evidenced by the lower specific surface area and inferior compressive strength of Geo-BT10/28d (28.8 MPa) compared with Geo-BT5/28d (35.7 MPa).

**Fig. 6:**
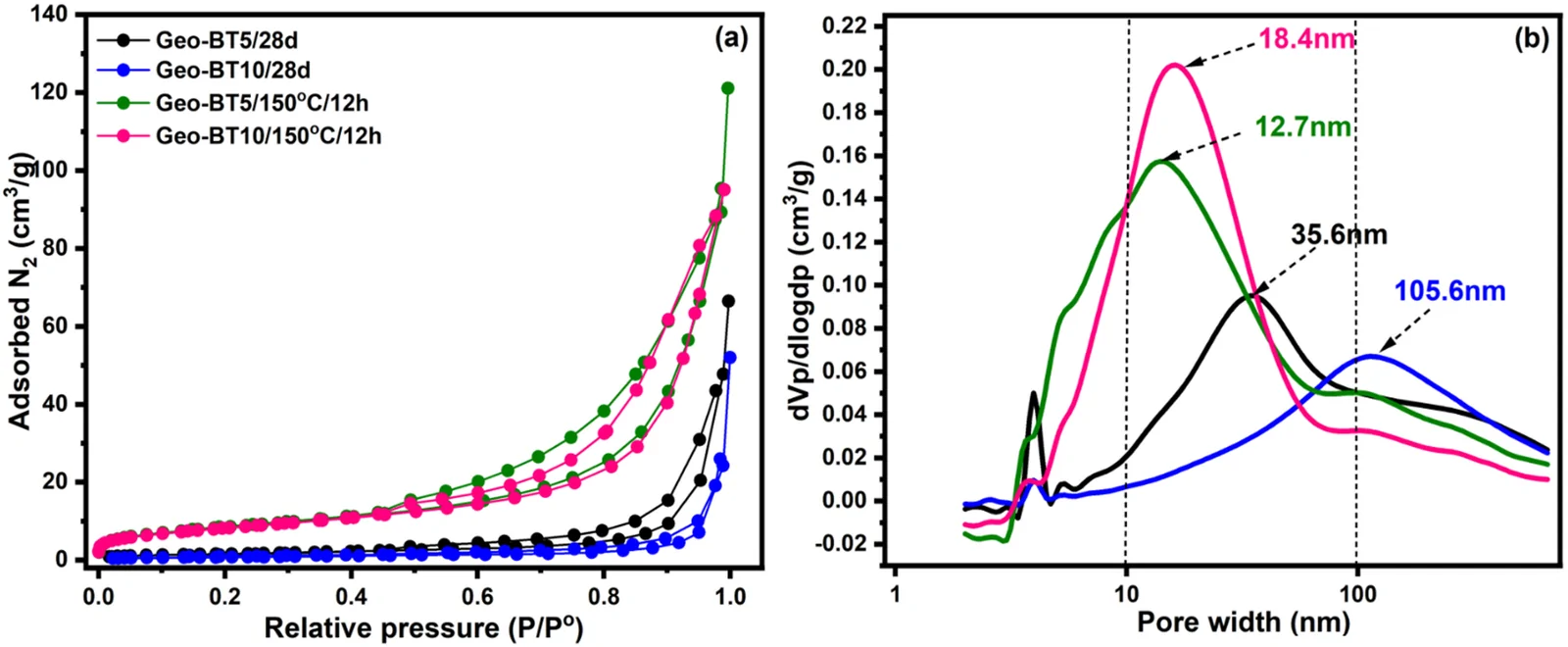
(a)N_2_-adsorption/desorption isotherms and (b) BJH-pore size distributions of Geo-BT5 and Geo-BT10 geopolymers fabricated under different curing regimes.

**Table 3:** Surface characterizations of Geo-BT5 and Geo-BT10 geopolymers prepared under different curing conditions

Composite	BET surface area (m^2^ g^−1^)	Total pore volume (*V*_p_, cm^3^ g^−1^)	Max pore diameter dp_max_ (nm)	Isotherm type
Geo-BT5/28d	5.94	0.078	35.6	III
Geo-BT10/28d	2.65	0.043	105.6	III
Geo-BT5/150 °C/12 h	30.91	0.158	12.7	IV/H3
Geo-BT10/150 °C/12 h	30.07	0.146	18.4	IV/H3

Similar results have been reported by Mohsen *et al.*^34^ and Chithambaram *et al.*,^69^ where alkali concentrations exceeding the optimum level resulted in non-uniform geopolymer gel formation, coarser pore structures, and reduced mechanical performance due to excessive dissolution and premature precipitation of aluminosilicate chains.

A significant modification of the pore structure occurred after autoclave curing at 150 °C/4.76 bar for 12 h. Both autoclaved samples exhibited Type IV adsorption isotherms with H3 hysteresis loops, characteristic of mesoporous materials containing slit-shaped pores. The BET surface areas increased markedly to 30.91 and 30.07 m^2^ g^−1^ for Geo-BT5/150 °C/12 h and Geo-BT10/150 °C/12 h, respectively, while the pore volumes increased to 0.158 and 0.146 cm^3^ g^−1^. Concurrently, the dominant pore diameters decreased dramatically to 12.7 nm (green curve) and 18.4 nm (pink curve), respectively. The transition from Type III to Type IV/H3 isotherms demonstrates the profound influence of hydrothermal curing on pore development and meso-structural refinement. The BJH pore size distributions clearly confirm the pore-refinement effect induced by autoclave curing. The maximum pore diameter decreased from 35.6 to 12.7 nm in Geo-BT5 and from 105.6 to 18.4 nm in Geo-BT10. These reductions correspond to approximately 64% and 83% decreases in characteristic pore size, respectively. The refinement process is schematically illustrated in Fig. 7, where large capillary pores are transformed into a denser network of finer mesopores.^34^ Under hydrothermal conditions, the enhanced dissolution of slag/basalt precursor facilitates the formation of additional highly crystalline hydrates (CSH, CASH),^9,70^ Fe-containing phases, in addition to zeolitic-like phases (analcime, gismondine, sodalite).^71,72^ These newly formed binding phases progressively fill and partition larger voids, generating a more homogeneous and compact microstructure.^36,67^ Autoclave curing significantly refined the pore structure of both geopolymer systems (Geo-BT5 and Geo-BT10); nevertheless, Geo-BT5/150 °C/12 h developed a markedly denser microstructure than Geo-BT10/150 °C/12 h. The former exhibited a dominant pore diameter of 12.7 nm, compared with 18.4 nm for the latter, indicating superior pore refinement. Despite the two autoclaved mixtures displaying nearly identical BET specific surface areas, the finer pore network observed in Geo-BT5 suggests that the 5% NaOH dosage promoted a more optimal balance among precursor dissolution, gel formation, and crystallization processes.

**Fig. 7:**
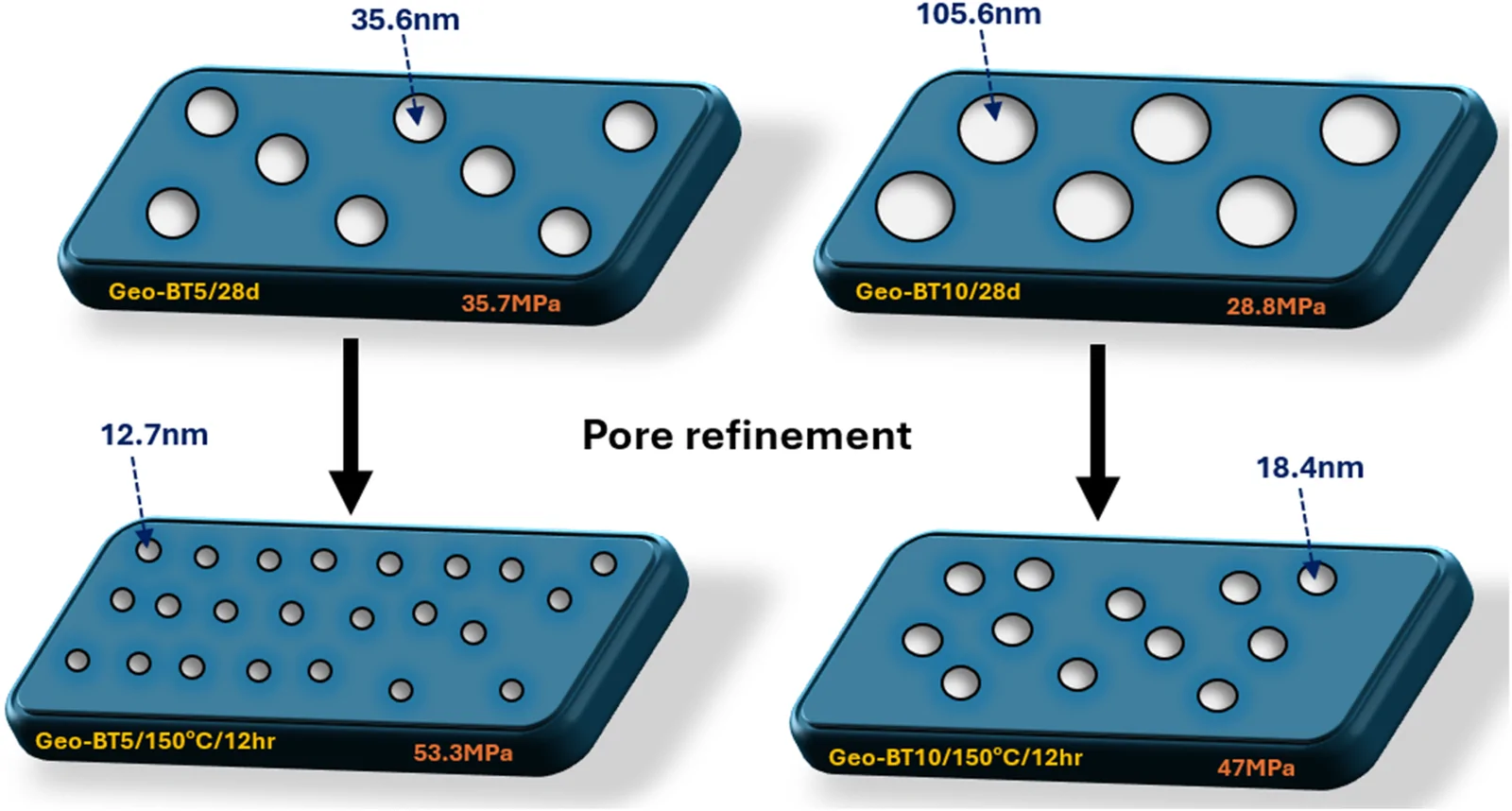
Conceptual diagram depicting pore refinement in fabricated geopolymeric specimens following autoclave curing.

The evolution of the pore structure was closely associated with the mechanical behavior of the geopolymer composites.^36^ As illustrated in Fig. 7, autoclave curing enhanced the compressive strength of Geo-BT5 from 35.7 to 53.3 MPa and that of Geo-BT10 from 28.8 to 47.0 MPa. The superior performance of Geo-BT5/150 °C/12 h, characterized by its high compressive strength (53.3 MPa), large specific surface area (30.91 m^2^ g^−1^), and highly refined mesoporous structure (12.7 nm), demonstrates that this material possesses functionality beyond conventional structural applications. In addition to providing excellent load-bearing capacity, its developed surface characteristics offer promising adsorption potential for water treatment purposes.^31,70^ This combination of structural and environmental functionalities enhances the overall sustainability and added value of the slag-basalt geopolymer system.

### 3.4 Phase identification and morphology

#### 3.4.1 X-ray diffraction analysis

The phase composition of the solid powders and the successful formation of the fabricated materials were determined by powder XRD.^73,74^ The XRD-patterns of Geo-BT5 developed composites under 28-days normal curing and 150 °C/12 h hydrothermal curing reveal significant differences in phase composition, reaction extent and microstructure development (Fig. 8). Such differences support strong evidence for the clear enhancement in the compressive-strength after hydrothermal curing; Geo-BT5/150 °C/12 h has a higher strength value than Geo-BT5/28-days by 49.3%. This confirms the role of hydrothermal curing in accelerating the activation of unreacted phases, increasing the reactivity of inactive precursors and hastening the geopolymerization/consolidation of GGBFS/BT-based matrix.

**Fig. 8:**
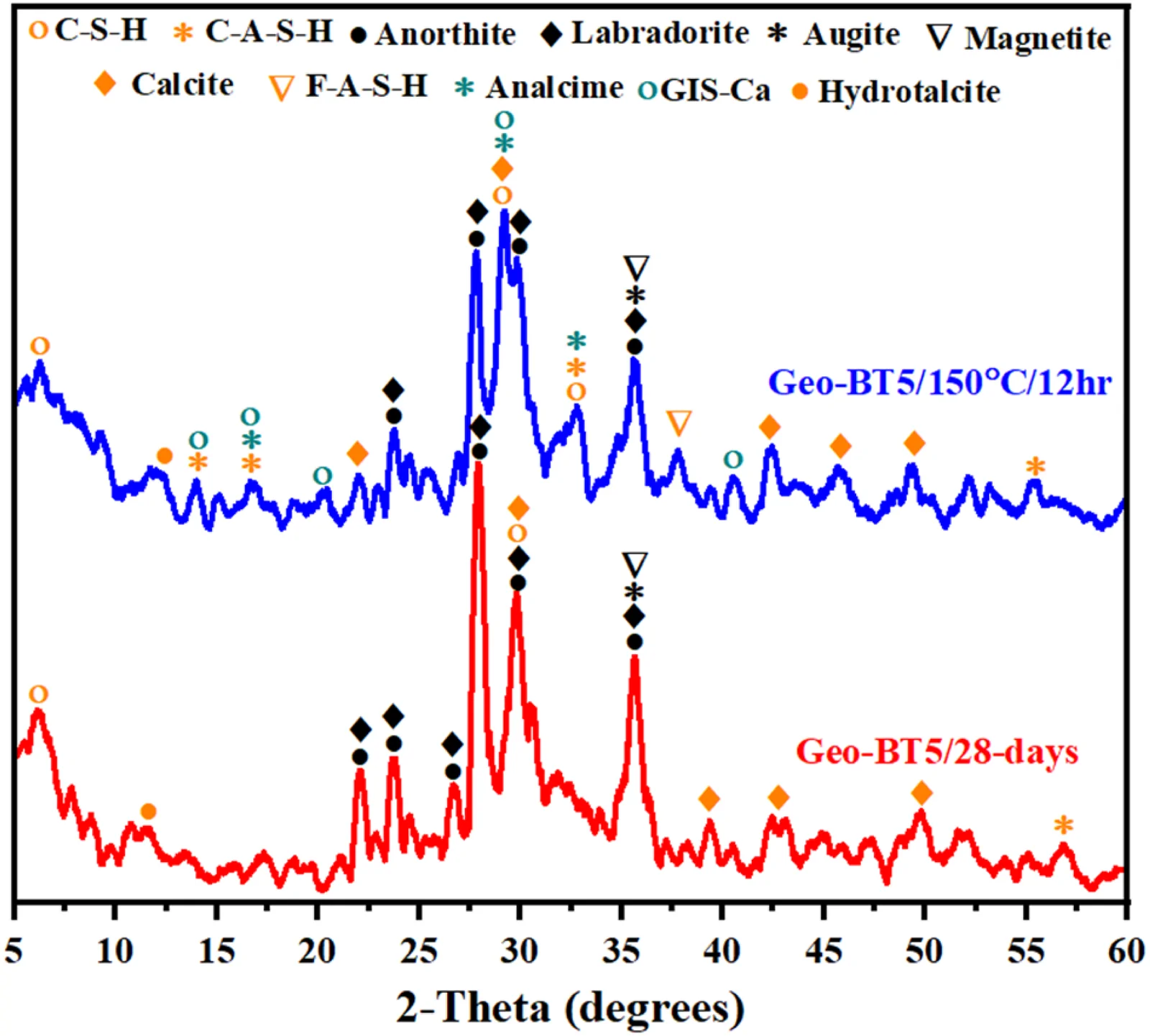
XRD-analysis of Geo-BT5 under different curing conditions.

Under normal curing conditions at 28-days, the diffractogram is dominated by a sharp peak affiliated with the residual crystalline BT phases. For instance, anorthite (CaAl_2_Si_2_O_8_, PDF# 96-100-0035) and labradorite (Ca_0.67_Al_1.67_Si_2.33_Na_0.33_O_8_, PDF# 96-210-8234), which are overlapped at 2*θ* = 22.19, 23.87, 26.63, 27.99, 29.85 and 35.77°. In addition, peaks associated with augite (Ca_0.6_Al_0.34_Si_1.82_Fe_0.2_Mg_0.9_Na_0.1_O_6_, PDF# 96-100-0036), as well as magnetite (Fe_3_O_4_, PDF# 96-101-1033) overlapped at 2*θ* = 35.77°. The existence of such phases indicates that a large amount of BT remains intact under normal curing, which is expected due to its high crystallinity, as well as its mineralogical nature, which is composed of a stable aluminosilicate and iron-bearing phases. The same observations were previously detected by Abd Al-Aziz *et al.*^48^ On the contrary, the unreacted phases of GGBFS had disappeared due to its high reactivity, which stems from its glassy nature. Furthermore, the XRD-pattern for Geo-BT5/28-days shows a clear broad band at 2*θ* = 25.00–37.00°, which is assigned to the formation of load-bearing capacity phases from calcium-silicate-hydrate (C–S–H, Ca_4.5_Si_6_O_15_(OH)_3_·2H_2_O, PDF# 00-019-0052) and calcium-alumino-silicate-hydrate (C-A-S-H, Ca_5_Si_5_Al(OH)O_17_·5H_2_O, PDF# 00-019- 0052). In addition, a weak diffraction peak corresponding to hydrotalcite (PDF# 00-014-0525) is observed at 2*θ* = 11.69°, further confirming the presence of secondary hydration products.^75^ However, the relatively broad and ill-crystalline peak characteristics of these phases provide for the formation of a semi-amorphous binding gel.^76^ Although such gels contribute to strength progression, the coexistence of unreacted BT indicates that the degree of the geopolymerization reactions at 28-days was not sufficient to densify the microstructure.

Under hydrothermal curing at 150 °C/12 h, it is worth noting that the intensity of the peaks related to the unreacted phases from BT (anorthite, labradorite, augite and magnetite) diminished, confirming the superior role of hydrothermal curing in enhancing the dissolution of stable phases.^65^ Simultaneously, the intensity of the peaks related to C-S-H and C-A-S-H becomes more significant and sharper, referring to the impact of hydrothermal curing in transforming the amorphous gel into cross-linked phases under steam pressure.^71^ The XRD-pattern of Geo-BT5/150 °C/12 h exhibits the formation of new zeolitic phases. Analcime (Al_1.81_H_4_Na_1.71_O_14_Si_4.194_, PDF#00-019-1180) at 2*θ* = 17.09, 29.11 and 32.73°, gismondine-Ca (GIS-Ca, CaAl_2_Si_2_O_8_·4H_2_O, PDF# 00-020-0452) at 2*θ* = 13.99, 17.09, 20.55, 29.11 and 40.67° and iron-alumino-silicate-hydrate (F-A-S-H, Fe_6.7_(Al_2_Si_3_O_10_)_8_(H_2_O)_30_), PDF# 01-090-8933) at 2*θ* = 37.73° are the new phases detected. The formation/intensification of these phases indicates that hydrothermal curing promotes the dissolution of aluminosilicate and Fe-bearing phases in GGBFS and BT, allowing precipitation of secondary strength-giving phases. Consequently, the matrix is transformed into more chemically integrated composites, including Ca-rich aluminosilicate hydrate and zeolitic-like phases, which fill pores/open-voids and refine the microstructure, as will be proved below by SEM and BET/BJH.

#### 3.4.2 Thermogravimetric analysis (TGA/DTGA)

Fig. 9a and b presents the TGA/DTGA thermograms of the Geo-BT5 composite paste subjected to different curing conditions: ambient curing for 28 days (Fig. 9a) and hydrothermal curing at 150 °C for 12 h (Fig. 9b). Under ambient curing conditions, the thermograms exhibit three successive mass-loss stages accompanied by four endothermic DTGA peaks. The first mass-loss stage (7.36 wt%) occurring at 50–300 °C is mainly attributed to the removal of physically adsorbed and bound water, along with the dehydration of strength-giving hydrates such as calcium silicate hydrates (CSHs), calcium aluminosilicate hydrates (CASHs), sodium aluminosilicate hydrate (NASH), and iron aluminosilicate hydrate (FASH) gels formed during the alkali activation of the slag/basalt binders.^77^ The second mass-loss stage (3.06 wt%), observed within the temperature range of 300–600 °C, is associated with the dehydroxylation of hydrotalcite-like phases (HT) and hydrogarnet (C_3_AH_6_).^9^ The final mass loss (3.85 wt%) occurring between 600 and 1000 °C is characterized by two distinct endothermic peaks and is primarily related to the decarbonation of carbonate-bearing phases, particularly calcite.^67^ The significantly lower mass loss of the hydrothermally cured paste (from 14.27% to 8.08%) reflects the formation of a more condensed and thermally stable geopolymeric networks. The development of crystalline zeolitic phases, namely analcime and gismondine (as clarified in XRD), contributed to matrix densification and microstructural refinement, leading to a substantial increase in compressive strength from 37.5 to 53.3 MPa compared with the normally cured counterpart. This inverse relationship between TGA mass loss and mechanical performance is in excellent agreement with the findings of Sayed *et al.*^65^ and Ramadan *et al.*,^11^ who reported that hydrothermal treatment reduced the TGA values while significantly enhancing the compressive strength of alkali-activated binders due to the development of thermally stable crystalline phases. In their study, the strength improvement was attributed to the formation of crystalline hydrates and zeolitic products, including analcime, chabazite, and zeolite-A.

**Fig. 9:**
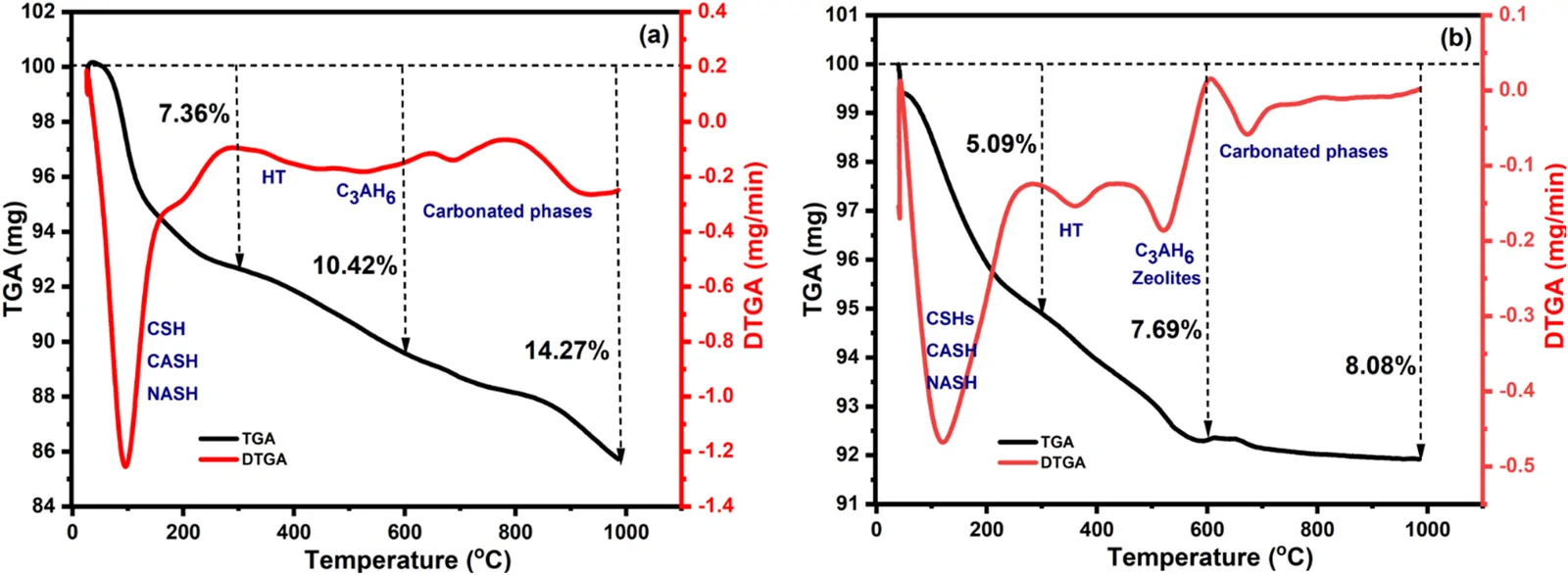
TGA/DTGA thermograms of Geo-BT5 under different curing conditions: (a) 28-days of normal curing and (b) hydrothermal curing at 150 °C/12 h.

#### 3.4.3 FTIR-spectra

Fig. 10 illustrates the FTIR spectra of Geo-BT5 geopolymer pastes subjected to two advanced curing regimes: ambient curing under high-humidity conditions at room temperature for 28 days (Geo-BT5/N) and hydrothermal curing *via* autoclaving at 150 °C/4.76 bar for 12 h (Geo-BT5/H), highlighting the influence of curing environments on the development of the hydration products (CSH, CASH) and geopolymeric structures (N-A-S-H, C(N)-A-S-H). The spectra display several characteristic absorption bands corresponding to the principal binding phases in addition to 3D-aluminosilicate networks. The bands in the 400–550 cm^−1^ region are attributed to the bending vibrations of Si–O–Si and Si–O–Al linkages,^78^ while the intense band located at 850–1250 cm^−1^ centered at 970 cm^−1^ corresponds to the asymmetric stretching vibrations of Si–O–Si/Si–O–Al bonds.^79^ The absorption peak near 1436 cm^−1^ is related to carbonate-containing phases or carbonation of Geo-BT pastes. Furthermore, the band around 1645 cm^−1^ is assigned to the bending vibration of molecular water (H–O–H), whereas the broad band at 3000–3600 cm^−1^ corresponds to the stretching vibration of hydroxyl (O–H) groups originating from adsorbed water and hydrated reaction products.^80^ Compared with normal curing, the autoclaved paste exhibited a more intense and sharper Si–O–Si(Al) stretching band in the 850–1250 cm^−1^ region, indicating a higher degree of aluminosilicate dissolution and polycondensation. This enhancement can be attributed to three main reasons: (i) the elevated temperature and pressure accelerated the dissolution of slag/basalt precursor, increasing the availability of geopolymeric networks; (ii) the hydrothermal environment promoted faster nucleation and growth/crystallization of C-S-H and C-A-S-H, resulting in a denser matrix;^36^ (iii) autoclave curing enhanced the condensation reactions between silicate and aluminate species, leading to a more highly cross-linked three-dimensional Si–O–Al frameworks and more ordered zeolitic phases,^80^ as confirmed by XRD, which contributed to structural stabilization and pore refinement. (iv) The slight displacement of the Si–O–M (M = Si, Al and Fe) vibration band from 953 to 966 cm^−1^ is indicative of a more advanced aluminosilicate framework development, highlighting the effective incorporation of Al and Fe into the network structure.^36,81^ The autoclaved paste exhibited lower intensities of the O–H (∼3450 cm^−1^) and H–O–H (∼1645 cm^−1^) bands, indicating the consumption of physically adsorbed and the progression of condensation reactions.^81^ The carbonate band near 1436 cm^−1^ was more pronounced in the Geo-BT5/N paste, which can be attributed to its prolonged exposure to atmospheric CO_2_,^80^ whereas the denser matrix (Geo-BT5/N) produced by autoclave curing restricted carbonation. Consequently, the accelerated reaction kinetics, enhanced formation of CSH and CASH, increased cross-linking, zeolitic phase development, and matrix densification produced a more compact geopolymeric structure, leading to an increase in compressive strength from 37.5 MPa to 53.3 MPa. Moreover, hydrothermal treatment promoted the generation of zeolitic and aluminosilicate active sites, improving the adsorption capacity of the Geo-BT5/H geopolymer and enhancing its dye removal performance. Abd El-Lateef *et al.*^36^ and Amin *et al.*^70^ reported that the dye removal performance of fabricated geopolymers is mainly governed by the presence of several surface functional groups and active sites within the geopolymeric chains. Surface hydroxyl groups associated with aluminosilicate gels (Si–OH, Al–OH) and hydration products (CSH, CASH) can interact with dye molecules through hydrogen bonding and electrostatic attraction. In addition, the Si–O–Al frameworks create negatively charged sites in the geopolymer network, providing ion-exchange and adsorption centers. The formation of zeolitic phases further increases the number of accessible adsorption sites and enhances meso-porosity of Geo-BT5/H facilitating stronger interactions between Geo-BT5/H and dye molecules.^71^

**Fig. 10:**
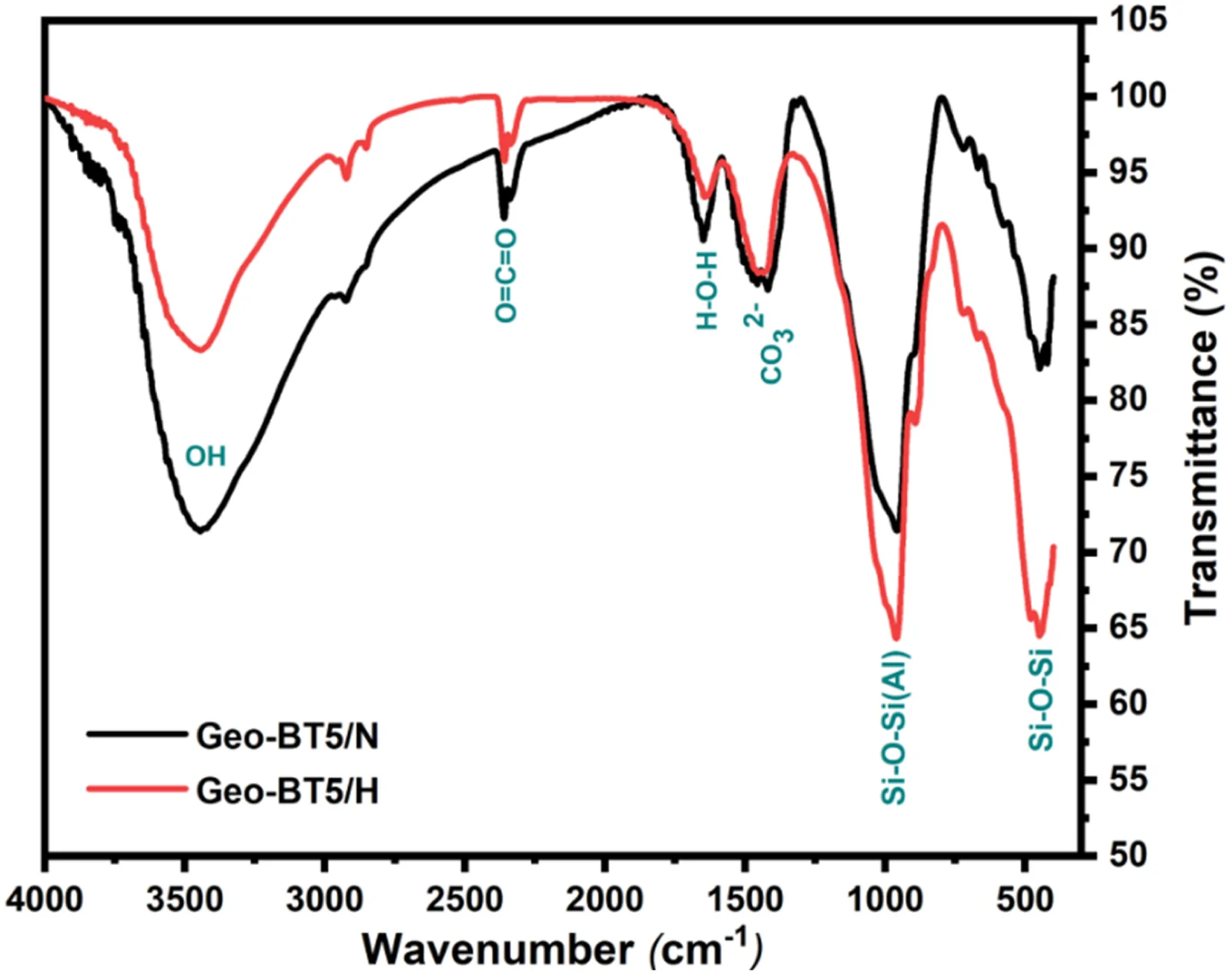
FTIR-spectra of Geo-BT5 pastes under different curing regimes.

#### 3.4.4 Morphology

SEM micrographs of Geo-BT5/28d, Geo-BT5/150 °C/12 h, and Geo-BT5/150 °C/24 h geopolymeric pastes are presented in Fig. 11(a1–c1), while the corresponding full-area EDX spectra are shown in Fig. 11(a2–c2). The microstructure of Geo-BT5/28d (Fig. 11a1) is characterized by a dense matrix composed mainly of calcium silicate hydrate (CSH) needles^9^ together with dispersed spherical hydrogarnet gel (CASH).^82^ The formed calcium-rich binding hydrates, resulting from the alkali activation of the slag/basalt blend, produced a relatively compact structure, corresponding to a compressive strength of 37.5 MPa after 28 days of ambient curing. In contrast, the microstructure of Geo-BT5/150 °C/12 h (Fig. 11b1) underwent a pronounced transformation following hydrothermal curing at 150 °C for 12 h. The needle CSH and spherical CASH were largely replaced by well-developed crystalline aggregates exhibiting plate-like and prismatic morphologies typical of zeolitic phases. Hydrothermal treatment promoted the dissolution of the aluminosilicate precursors and their subsequent reprecipitation and recrystallization into more thermodynamically stable aluminosilicate phases, resulting in the formation of highly ordered crystals.^36^ The development and intergrowth of zeolitic structures, including calcium zeolites (gismondine-type phases),^83^ sodalite-type phases,^84,85^ or ZSM-5 zeolites (analcime),^86,87^ produced a denser and more compact matrix with enhanced mechanical integrity. Consequently, the compressive strength increased significantly to 53.3 MPa, representing an improvement of approximately 42% compared with the conventionally cured specimen. Further extending the hydrothermal curing duration to 24 h at 150 °C resulted in noticeable changes in the microstructure of the geopolymeric paste (Fig. 11c1). Compared with the compact and highly intergrown zeolitic framework developed after 12 h of hydrothermal treatment, the 24 h specimen exhibited a less homogeneous microstructure characterized by the reappearance of abundant spherical hydrogarnet particles, dispersed zeolitic crystals, and the development of numerous microcracks throughout the matrix. The prolonged hydrothermal exposure likely promoted excessive dissolution-recrystallization reactions and crystal growth, leading to volumetric instability and the generation of internal stresses. As a result, localized cracking occurred, disrupting the continuity of the previously dense zeolitic network.^9^

**Fig. 11:**
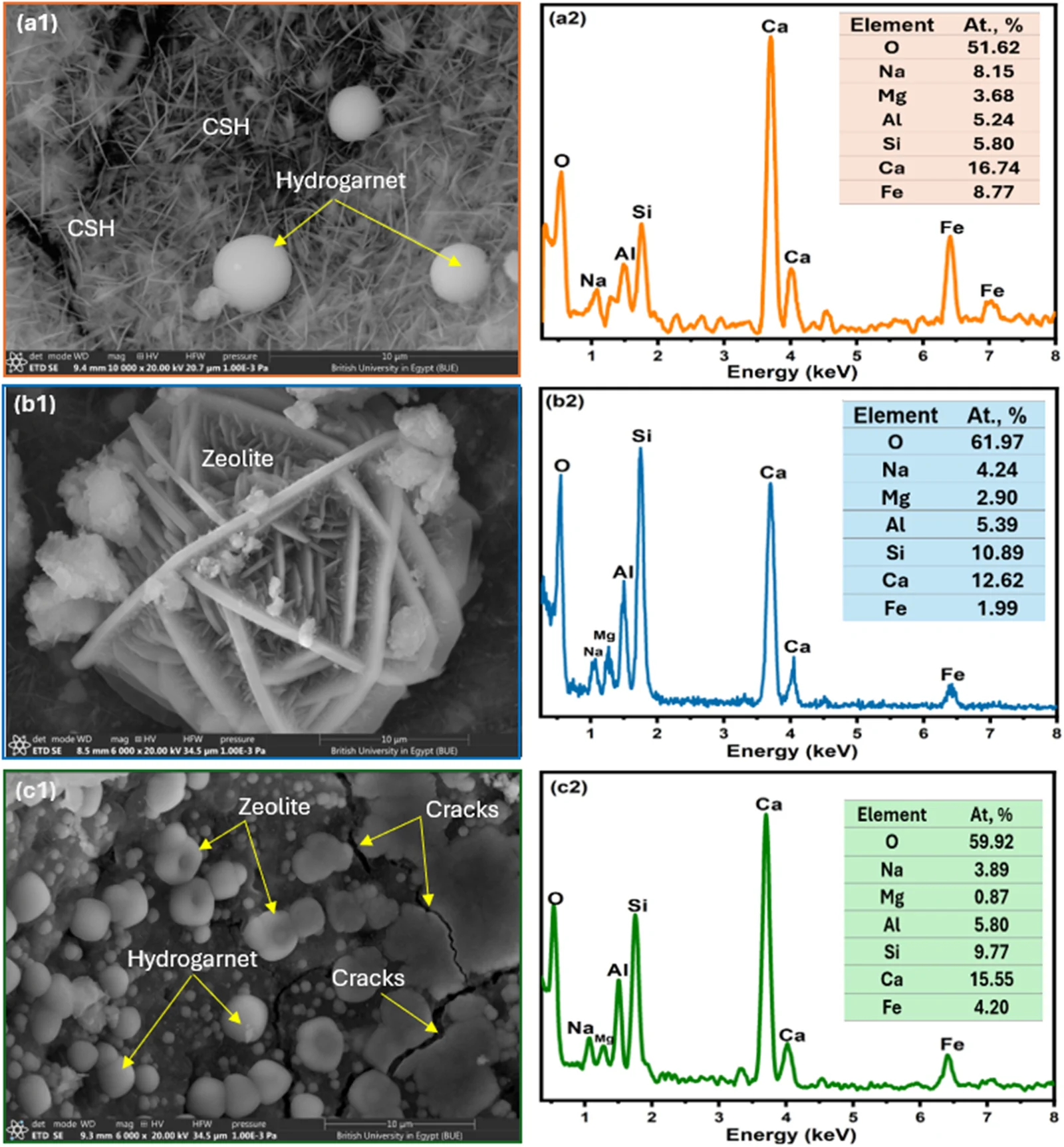
SEM/full-area EDX images of geopolymeric pastes: (a1 and a2) Geo-BT5/28 d, (b1 and b2) Geo-BT5/150 °C/12 h, and (c1 and c2) Geo-BT5/150 °C/24 h.

The full-area EDX analysis further supports the observed microstructural evolution. For Geo-BT5/28d (Fig. 11a2), the average composition was characterized by Ca (16.74%), Si (5.80%) and Al (5.24%) yielding a relatively high Ca/Si ratio of 2.89 and a Si/Al ratio of 1.11. These values are consistent with the predominance of calcium-rich phases (CSH and CASH). After autoclaving, substantial compositional changes were observed in Geo-BT5/150 °C/12 h (Fig. 11b2). The Si content increased markedly from 5.80 to 10.89%, while the Ca content decreased from 16.74 to 12.62%, resulting in a significant reduction in the Ca/Si ratio from 2.89 to 1.16. Simultaneously, the Si/Al ratio increased from 1.11 to 2.02, indicating the development of a more silica-rich and polymerized aluminosilicate framework. In addition, the Fe content decreased from 8.77 to 1.99%, suggesting limited incorporation of iron into the newly formed crystalline products. The combined SEM and EDX results demonstrate that hydrothermal curing promoted recrystallization of hydration products into Ca-bearing zeolitic phases through a dissolution-reprecipitation mechanism. The marked decrease in the Ca/Si ratio, together with the increase in Si/Al ratio indicates enhanced crystallization of hydration yields into more ordered zeolitic aluminosilicate frameworks. This phase evolution contributed significantly to matrix densification and is considered the main reason for the considerable enhancement in compressive strength observed after autoclave curing. The EDX results of Geo-BT5/150 °C/24 h (Fig. 11c2). revealed a slight increase in Ca content (15.55%) and a decrease in Si content (9.77%) compared with the 12 h autoclaved specimen, resulting in an increase in the Ca/Si ratio from 1.16 to 1.59 and a decrease in the Si/Al ratio from 2.02 to 1.68. These changes indicate partial transformation of the highly polymerized silica-rich zeolitic framework formed after 12 h into more calcium-rich crystalline phases, consistent with the reappearance of hydrogarnet and the development of microcracks observed in the SEM image. Consequently, the compressive strength decreased from 53.3 MPa to 47.5 MPa, although it remained higher than that of the ambient-cured sample (37.5 MPa). These findings suggest that 12 h of hydrothermal curing at 150 °C represents the optimum condition for achieving maximum matrix densification and mechanical performance. According to Ma *et al.*,^88^ an increase in the Si/Al ratio to 1.75–2.0 promotes the conversion of zeolitic phases into analcime, which becomes the dominant crystalline phase. Liang *et al.*^89^ reported that decreasing the Ca/Si ratio promotes analcime formation, whereas higher Ca/Si ratios favor the development of C-A-S-H gel and tobermorite.

### 3.5 Dye removal application

The hydrothermally cured geopolymer Geo-BT5/H was selected for adsorption studies because it exhibited the most favorable combination of mechanical performance, economic feasibility, and pore characteristics among the fabricated materials. Compared with the normally cured specimens, hydrothermal treatment generated a highly developed mesoporous network together with abundant aluminosilicate and zeolitic active sites, which are expected to facilitate mass transfer and provide a greater number of adsorption centers for dye molecules.^90^ The improved textural properties, together with the high compressive strength, enable Geo-BT5/H (autoclaving at 150 °C/12 h) to function as a durable and reusable adsorbent for wastewater treatment without compromising its structural stability.

#### 3.5.1 Effect of pH

The solution pH is one of the most influential parameters governing adsorption because it simultaneously controls the surface charge of the adsorbent and the ionization state of the adsorbate.^43,91^Fig. 12A demonstrates that the adsorption efficiency of Indigo Carmine (IC) strongly depends on the solution pH, with the maximum removal occurring under strongly acidic conditions (approximately pH 2), followed by a continuous decline as the pH increases. This behavior can be interpreted according to the point of zero charge (pHpzc) of the developed geopolymer (Fig. 12B). The pHpzc of Geo-BT5/H is approximately 8.5 (according to the presented curve). At solution pH values below pHpzc, the hydroxyl groups present on the geopolymer surface (Si–OH, Al–OH and Fe–OH) become protonated, generating positively charged adsorption sites.^92^ The positively charged surface strongly attracts the negatively charged sulfonate groups (–SO_3_^−^) of indigo carmine through electrostatic attraction, resulting in high adsorption efficiency. Conversely, at pH values exceeding the pHpzc, surface hydroxyl groups undergo deprotonation which renders the geopolymer surface negatively charged.^93^ Since indigo carmine remains predominantly anionic over neutral and alkaline pH ranges, electrostatic repulsion develops between the negatively charged dye molecules and the negatively charged adsorbent surface, causing a progressive decrease in adsorption efficiency.^94^

**Fig. 12:**
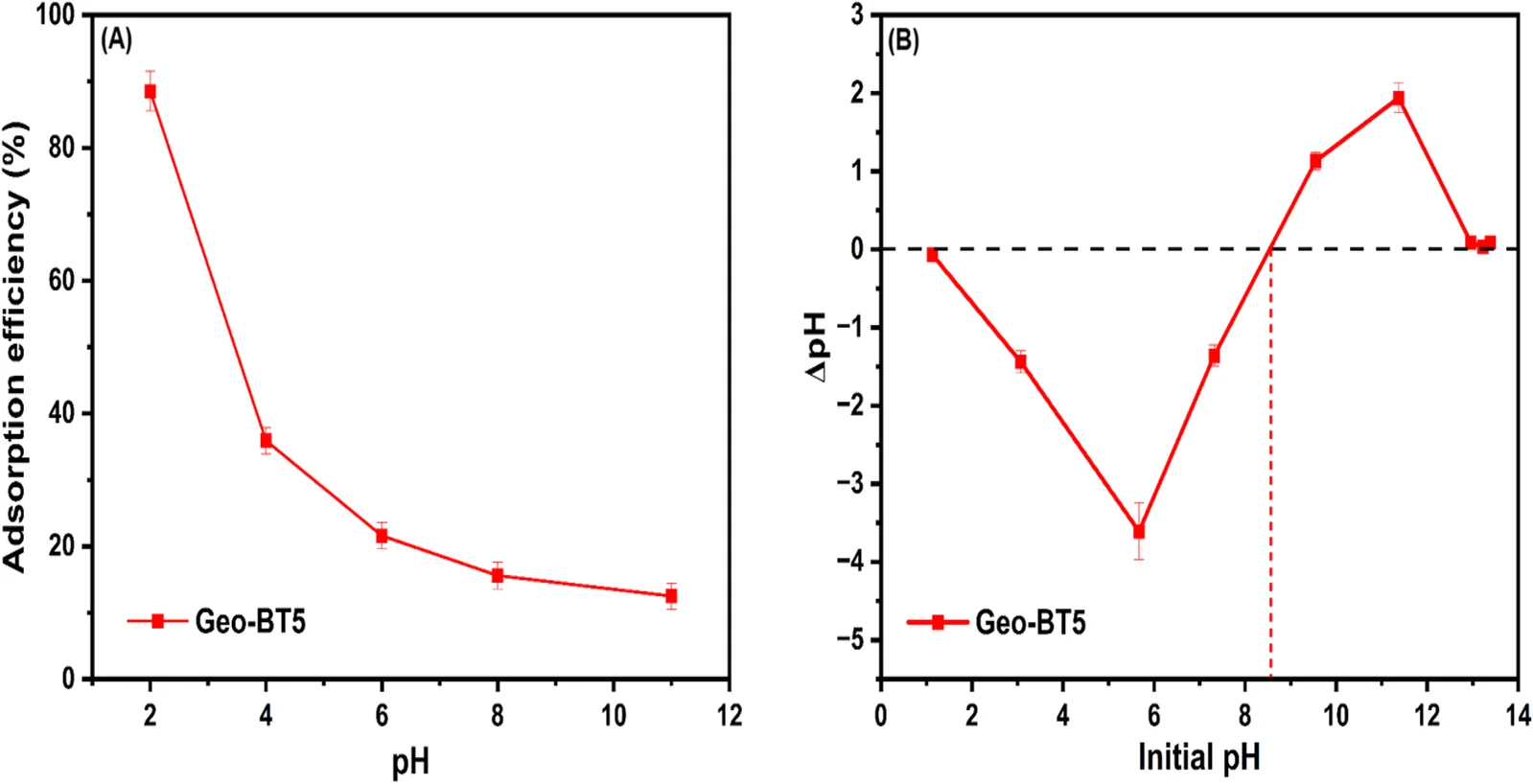
(A) Effect of solution pH on the adsorption of IC dye onto Geo-BT5/H (contact time = 120 minutes, temperature = 298 K, concentration of IC = 20 mg L^−1^, volume = 50 mL, adsorbent dose = 0.4 g), (B) point of zero charge (pzc) of the developed Geo-BT5/H adsorbent.

Besides electrostatic interactions, hydrogen bonding between surface hydroxyl groups and oxygen-containing functional groups of the dye, together with pore filling within the mesoporous geopolymer framework, also contributes to dye uptake. Nevertheless, electrostatic attraction appears to be the dominant adsorption mechanism under acidic conditions. To further improve adsorption under acidic media, the geopolymer was subjected to acid activation using HCl. The BET analysis after acid treatment (pH ∼2) (Fig. 13 and Table 4) revealed that the specific surface area increased from 30.91 to 39.7 m^2^ g^−1^, while the total pore volume increased to 0.171 cm^3^ g^−1^ and the average pore diameter slightly decreased to 11.61 nm, maintaining the characteristic Type IV/H3 mesoporous structure. The improvement in textural properties after acid treatment can be attributed to the partial dissolution of loosely bound calcium-rich phases and the removal of impurities partially blocking the pore channels. This process exposes additional active adsorption sites and increases pore accessibility without destroying the mesoporous geopolymer framework.^36^ Consequently, acid activation further enhances the adsorption performance of Geo-BT5/H toward anionic indigo carmine. Although the maximum IC adsorption was achieved under strongly acidic conditions (pH ≈ 2), this condition should be regarded as the optimum experimental condition rather than a universal requirement for practical wastewater treatment; therefore, further optimization under less acidic or near-neutral conditions and evaluation using real wastewater are necessary to establish the practical and economic feasibility of Geo-BT5/H for large-scale application.

**Fig. 13:**
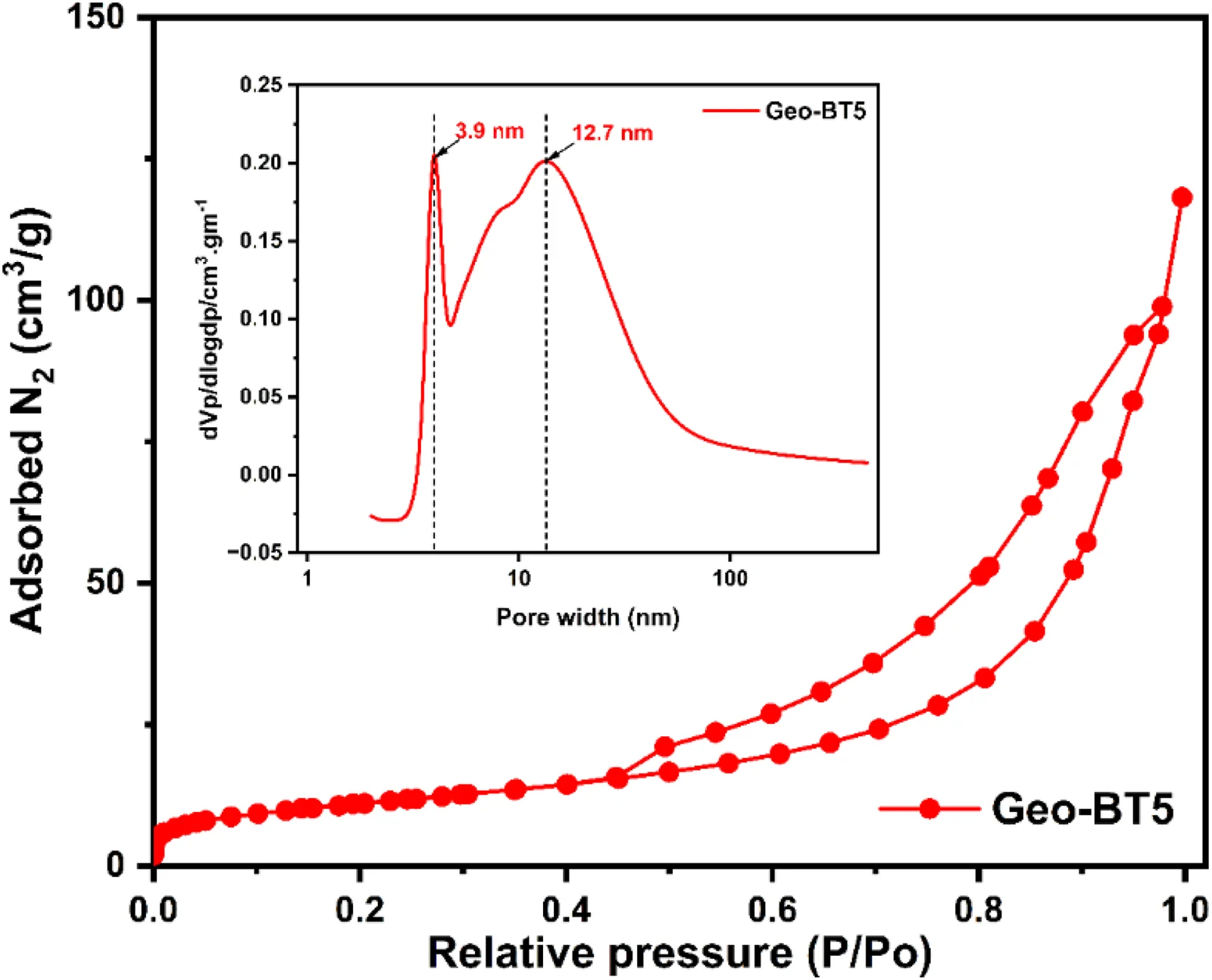
N_2_ adsorption/desorption isotherms with pore size distribution (PSD; inset) of the developed Geo-BT5/H adsorbent.

**Table 4:** Textural parameters for Geo-BT5/H under acidic treatment (pH ∼ 2)

Adsorbent	BET surface area (m^2^ g^−1^)	Total pore volume (*V*_p_, cm^3^ g^−1^)	Average pore diameter (nm)	Isotherm type
Geo-BT5/H	39.7	0.171	11.61	IV/H3

#### 3.5.2 Effect of temperature

The influence of temperature on indigo carmine adsorption is presented in Fig. 14A. The adsorption capacity gradually increases with increasing temperature from 293 to 353 K, indicating that the adsorption process is thermally promoted. Several factors contribute to this behavior. Increasing temperature enhances the mobility of dye molecules in solution, thereby reducing mass-transfer resistance and facilitating diffusion through the mesoporous channels of Geo-BT5/H.^95^ Elevated temperatures also accelerate intraparticle diffusion, allowing dye molecules to reach adsorption sites located deeper within the geopolymer framework.^96^ Furthermore, increasing temperature may activate previously inaccessible adsorption sites by increasing the flexibility of the aluminosilicate framework and reducing hydration barriers surrounding the surface functional groups.^97^ These effects collectively increase the equilibrium adsorption capacity. The linear Van't Hoff relationship shown in Fig. 14B indicates that the adsorption process follows classical thermodynamic behavior over the investigated temperature range, allowing reliable determination of Δ*H*°, Δ*S*°, and Δ*G*°.

**Fig. 14:**
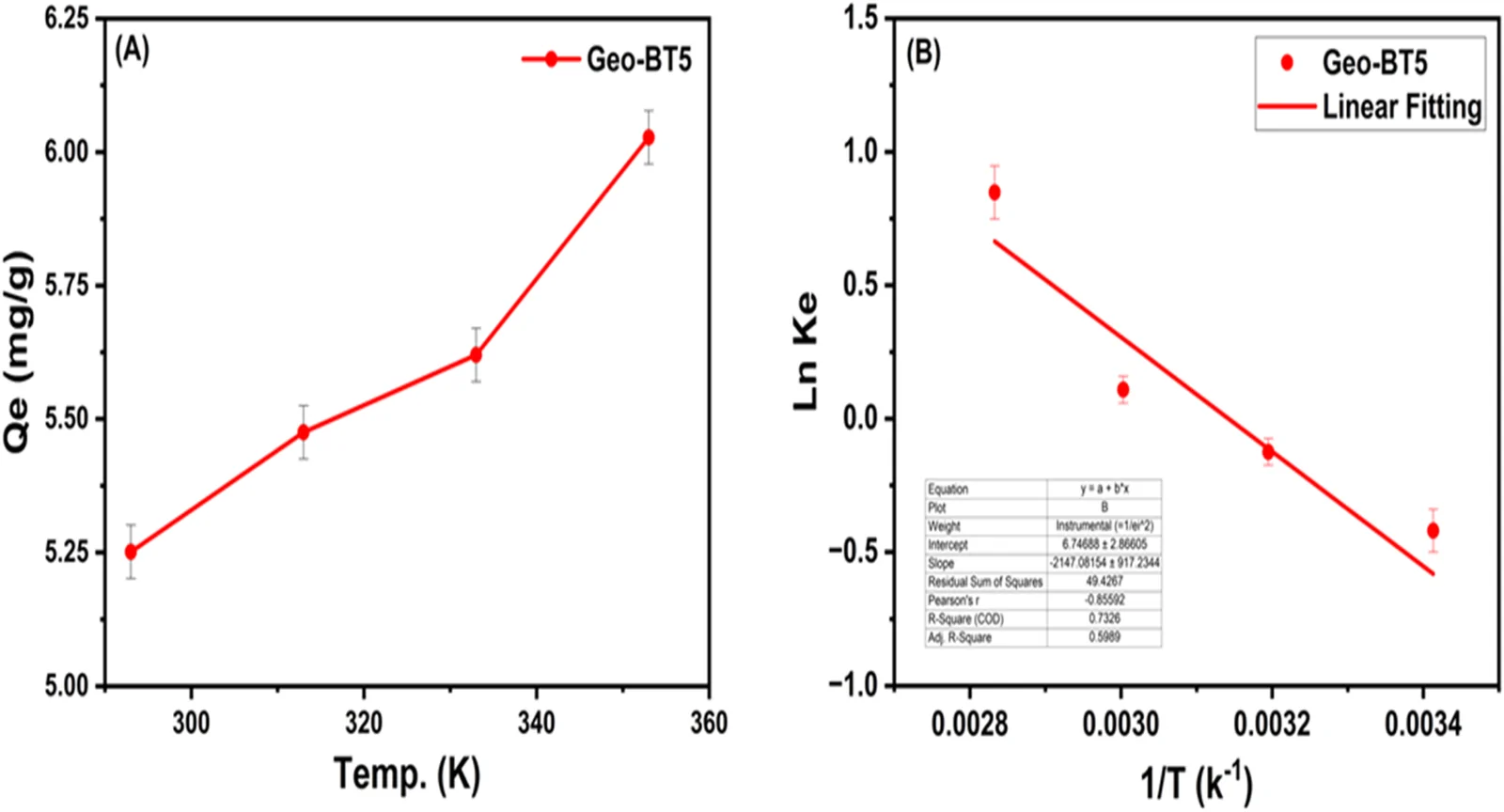
(A) Effect of operating temperature on the adsorption of IC dye onto the developed adsorbent, (B) Van't Hoff plots of ln *K*_e_*versus* (1/*T*) for IC dye adsorption onto Geo-BT5/H (contact time = 120 minutes, volume = 50 mL, adsorbent dose = 0.4 g, pH ≈ 2).

#### 3.5.3 Thermodynamic studies

The thermodynamic parameters calculated from the Van't Hoff equation are summarized in Table 5. The positive enthalpy change (Δ*H*° = 17.85 kJ mol^−1^) demonstrates that adsorption is an endothermic process. Accordingly, increasing the solution temperature favors adsorption, consistent with the experimental observations presented in Fig. 14. The magnitude of Δ*H*° is relatively low (<40 kJ mol^−1^), suggesting that the adsorption predominantly proceeds through physical interactions rather than strong chemical bond formation.^98,99^ These interactions mainly include electrostatic attraction, hydrogen bonding, van der Waals forces, and pore filling within the mesoporous geopolymer structure. The positive entropy change (Δ*S*° = 56.12 J mol^−1^ K^−1^) indicates increased randomness at the solid-solution interface during adsorption. This increase in entropy is commonly attributed to displacement of water molecules previously adsorbed on the geopolymer surface by indigo carmine molecules.^100,101^ The release of these structured water molecules into the bulk solution increases the overall disorder of the adsorption system. The Gibbs free energy values become progressively more negative with increasing temperature, changing from +1.44 kJ mol^−1^ at 293 K to −1.92 kJ mol^−1^ at 353 K. This trend indicates that adsorption becomes increasingly spontaneous as temperature rises. The slightly positive Δ*G*° at 293 K suggests that adsorption is only marginally favorable at room temperature, whereas the negative values observed above 313 K confirm the spontaneous nature of the process at elevated temperatures. Overall, the thermodynamic parameters demonstrate that indigo carmine adsorption onto Geo-BT5/H is an endothermic, entropy-driven, and predominantly physisorption-controlled process.

**Table 5:** Thermodynamic parameters for the adsorption of IC dye onto Geo-BT5/H adsorbent

Adsorbent	Δ*H*° (kJ mol^−1^)	Δ*S*° (J mol^−1^ K^−1^)	Δ*G*° (kJ mol^−1^)			
			293 K	313 K	333 K	353 K
Geo-BT5/H	17.85	56.12	1.44	0.322	−0.798	−1.918

#### 3.5.4 Effect of the initial dye concentration

Fig. 15A illustrates the influence of the initial indigo carmine concentration on adsorption by Geo-BT5/H. As the dye concentration increases, the adsorption capacity (mg g^−1^) increases continuously, whereas the percentage removal gradually decreases. At relatively low dye concentrations, the number of available adsorption sites greatly exceeds the number of dye molecules present in solution. Consequently, most dye molecules readily occupy active sites on the geopolymer surface, leading to high removal efficiency. As the initial dye concentration increases, the driving force for mass transfer between the solution and the adsorbent becomes larger, promoting diffusion of dye molecules into the internal mesoporous structure.^102^ This enhanced concentration gradient increases the adsorption capacity because a larger amount of dye becomes accumulated on the adsorbent. However, the total number of adsorption sites remains constant for a fixed adsorbent dosage. At elevated dye concentrations, progressive occupation of these active sites eventually leads to surface saturation. Once most adsorption sites have been occupied, additional dye molecules remain in solution, resulting in a gradual decline in percentage removal despite the increase in adsorption capacity.^103^ The excellent adsorption performance observed even at relatively high dye concentrations reflects the highly accessible mesoporous network developed during hydrothermal curing. The interconnected slit-shaped mesopores facilitate rapid intraparticle diffusion, while the abundant aluminosilicate and zeolitic active sites provide numerous adsorption centers capable of accommodating relatively large quantities of indigo carmine molecules.^104^

**Fig. 15:**
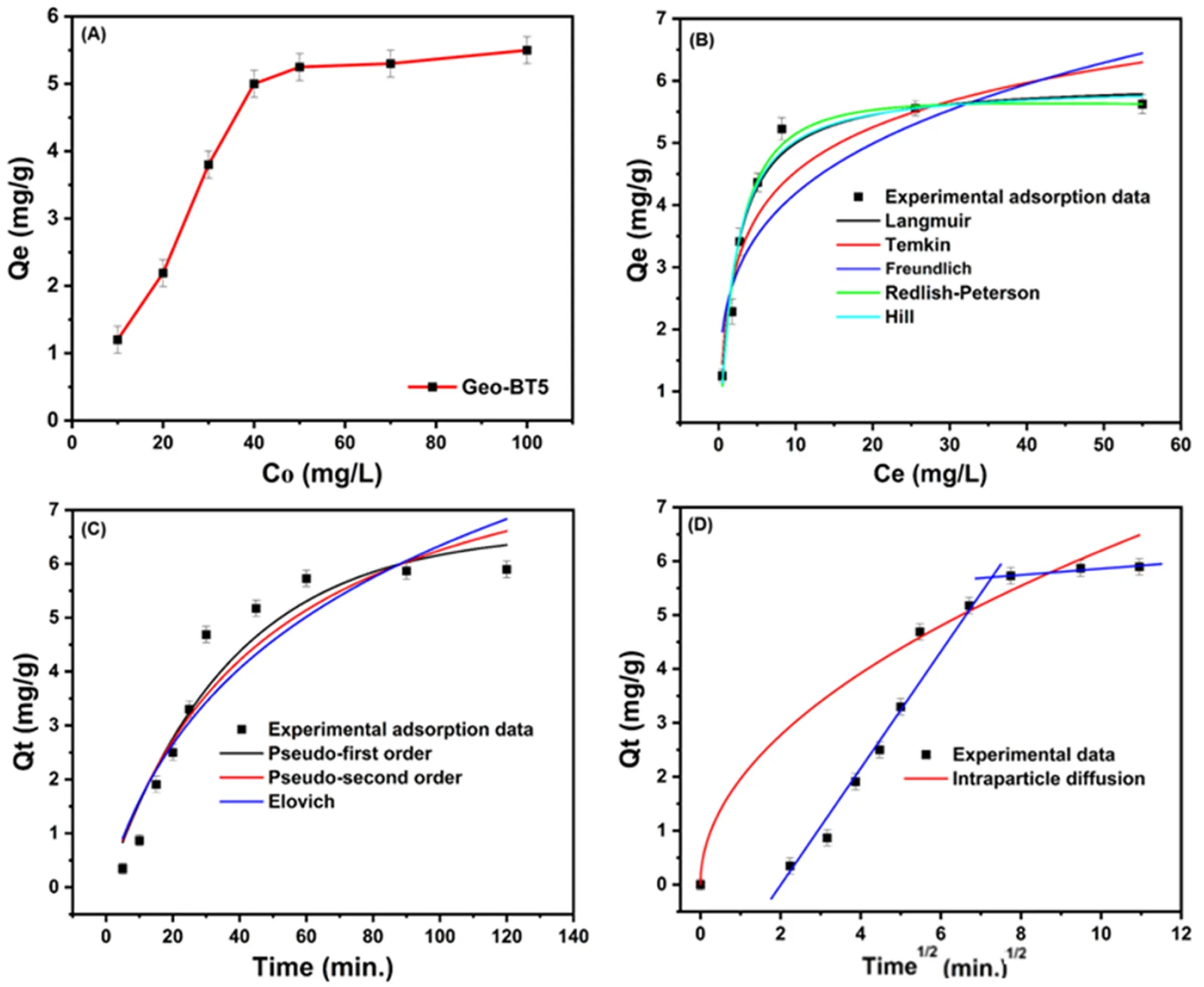
(A) Effect of initial concentration of dye on the adsorption of IC dye onto the developed Geo-BT5/H sorbent (contact time = 120 minutes, temperature = 293 K, volume = 50 mL, adsorbent dose = 0.4 g, pH ≈ 2), (B) nonlinear fitting plots of the Langmuir, Freundlich, Temkin, Redlich–Peterson, and Hill adsorption isotherm models, (C) nonlinear fitting plots of pseudo-first-order, pseudo-second order, and Elovich kinetic models, (D) intraparticle diffusion for the adsorption of IC dye on Geo-BT5/H.

#### 3.5.5 Adsorption isotherms

Adsorption isotherm analysis provides important information regarding the interaction between Indigo Carmine (IC) molecules and the adsorption sites of Geo-BT5/H at equilibrium. In the present study, the experimental equilibrium data were analyzed using five nonlinear isotherm models, namely Langmuir, Freundlich, Temkin, Redlich–Peterson, and Hill models, as shown in Fig. 15B. The corresponding fitting parameters and statistical indicators are summarized in Table 6. The Langmuir model assumes homogeneous adsorption sites and monolayer adsorption of adsorbate molecules onto a finite number of energetically equivalent active sites.^43,91^ As shown in Fig. 15B, the Langmuir equation provides a relatively good representation of the experimental data, with *R*^2^ = 0.988 and a calculated maximum adsorption capacity (*Q*_max_) of approximately 5.99 mg g^−1^. The good agreement indicates that monolayer-type occupation of adsorption sites constitutes an important component of IC adsorption onto Geo-BT5/H. Nevertheless, the deviation of the experimental points from the Langmuir curve, particularly in the intermediate concentration region, suggests that the surface cannot be considered completely homogeneous. The Freundlich model, which describes adsorption on energetically heterogeneous surfaces with a distribution of adsorption energies, exhibited a comparatively lower correlation coefficient (*R*^2^ = 0.851). The visibly greater deviation between the Freundlich curve and the experimental data in Fig. 15B further indicates that the conventional Freundlich equation does not adequately describe the complete equilibrium adsorption behavior of IC on Geo-BT5/H. This finding suggests that although surface heterogeneity exists, the adsorption process cannot be satisfactorily represented solely by the empirical Freundlich relationship. The Temkin model considers adsorbate–adsorbent interactions and assumes that the heat of adsorption decreases linearly with increasing surface coverage because of interactions between adsorbed molecules. The Temkin fitting curve provides a reasonable description of the experimental data, particularly over the intermediate concentration range. However, its overall agreement is inferior to that obtained using the Redlich–Peterson, Hill, and Langmuir models, indicating that a simple linear decrease in adsorption energy with increasing surface coverage does not fully describe the equilibrium behavior. In contrast, the Redlich–Peterson model provided the best overall statistical description of the experimental equilibrium data, exhibiting the highest correlation coefficient (*R*^2^ = 0.992) and the lowest chi-square value (*χ*^2^ = 2.36) among the investigated models. As visually demonstrated in Fig. 15B, the Redlich–Peterson curve closely follows the experimental data throughout the investigated concentration range. The superior fitting performance of this three-parameter model indicates that the equilibrium adsorption behavior incorporates characteristics of both the Langmuir and Freundlich models. In other words, the adsorption process exhibits appreciable monolayer adsorption characteristics while simultaneously reflecting a degree of surface heterogeneity. The Hill model also showed an excellent correlation with the experimental data (*R*^2^ = 0.989; *χ*^2^ = 3.04), indicating possible cooperative interactions among adsorbed IC molecules and/or adsorption sites. The close agreement between the Hill and Redlich–Peterson models further supports the existence of non-ideal adsorption behavior on the geopolymer surface. The comparable adsorption capacities predicted by the Hill model (approximately 5.92 mg g^−1^) and Langmuir model (5.99 mg g^−1^) provide additional consistency in the estimated equilibrium adsorption capacity. Overall, the statistical and graphical analyses indicate that the Redlich–Peterson model provides the most appropriate mathematical representation of the equilibrium adsorption data, followed closely by the Hill and Langmuir models. The predominance of these models suggests that IC adsorption onto Geo-BT5/H involves a combination of monolayer-type surface occupation and heterogeneous adsorption interactions rather than adsorption onto a perfectly homogeneous surface. This behavior is consistent with the structurally complex nature of the hydrothermally cured geopolymer, which contains different aluminosilicate, calcium-containing, and zeolitic phases together with hydroxylated surface sites and a developed mesoporous network. The equilibrium adsorption capacity obtained from the Langmuir model (∼5.99 mg g^−1^) also demonstrates the ability of Geo-BT5/H to accommodate IC molecules through its accessible adsorption sites. Considering that the material was developed from low-cost mineral precursors and simultaneously exhibits high mechanical strength and a well-developed mesoporous structure, its adsorption behavior provides an additional functional advantage for potential wastewater-treatment applications.

**Table 6:** Parameters of the adsorption isotherms for IC dye onto Geo-BT5/H

Model	Parameters	Value of parameters
Langmuir	*q*_m_ (mg g^−1^)	5.99
	*K*_L_ (L mg^−1^)	0.49
	*R* ^2^	0.988
	*χ* ^2^	2.54
Freundlich	*K*_F_	2.34
	*n*	3.95
	*R* ^2^	0.851
	*χ* ^2^	34.19
Temkin	*K*_T_ (L g^−1^)	8.03
	*B*_T_ (KJ mol^−1^)	1.034
	*R* ^2^	0.942
	*χ* ^2^	13.34
Redlich–Peterson	*K*_RP_ (L g^−1^)	2.54
	*A*_RP_ (L mg^−1^)	0.347
	*g*	1.0
	*R* ^2^	0.992
	*χ* ^2^	2.36
Hill	*q*_m_ (mg g^−1^)	5.922
	*K*_H_ (mg L^−1^)	2.02
	*n*	1.0
	*R* ^2^	0.989
	*χ* ^2^	3.04

#### 3.5.6 Kinetic studies

Adsorption kinetics provide insight into the rate of IC uptake and help identify the possible rate-controlling steps involved in the adsorption process. In the present study, the experimental kinetic data were analyzed using the pseudo-first-order (PFO), pseudo-second-order (PSO), Elovich, and intraparticle-diffusion models. The nonlinear fitting curves are presented in Fig. 15C and D, while the corresponding kinetic parameters are summarized in Table 7. As shown in Fig. 15C, the adsorption of IC onto Geo-BT5/H proceeds rapidly during the initial stage of the process, followed by a progressively slower increase in adsorption capacity until an approximately equilibrium condition is reached after about 120 min. The rapid initial uptake can be attributed to the abundance of readily accessible adsorption sites on the external surface of Geo-BT5/H and the large concentration gradient between the solution and the adsorbent at the beginning of the adsorption process. As these readily accessible sites become progressively occupied, the adsorption rate decreases and the remaining IC molecules must diffuse toward less accessible sites within the mesoporous structure. The pseudo-first-order model provided the highest correlation coefficient among the investigated kinetic models, with *R*^2^ = 0.941 and a calculated equilibrium adsorption capacity of approximately 6.60 mg g^−1^. As shown in Fig. 15C, the PFO curve provides a relatively close representation of the experimental adsorption profile, particularly during the later stage of adsorption. This relatively good fitting suggests that physical adsorption and mass-transfer processes associated with the availability of adsorption sites contribute significantly to the overall adsorption kinetics. The pseudo-second-order model also provided a satisfactory description of the kinetic data, with *R*^2^ 0.922 and a calculated equilibrium adsorption capacity of approximately 9.25 mg g^−1^. However, its correlation coefficient was lower than that obtained from the pseudo-first-order model, and the calculated equilibrium capacity deviated more substantially from the experimentally observed equilibrium capacity. Therefore, based on the present nonlinear fitting results, the pseudo-second-order model does not provide the most appropriate overall description of the experimental kinetic data.

**Table 7:** Kinetic parameters and their correlation coefficients for the adsorption of IC dye onto Geo-BT5/H

Model	Parameters	Value of parameters
Pseudo-first order	*k*_1_ (min^−1^)	0.027
	*q*_e_ (mg g^−1^)	6.60
	*R* ^2^	0.941
	*χ* ^2^	19.48
Pseudo-second order	*k*_2_ (g mg^−1^ min^−1^)	0.002
	*q*_e_ (mg g^−1^)	9.25
	*R* ^2^	0.922
	*χ* ^2^	25.59
Elovich	*α* (mg g^−1^ min^−1^)	0.21
	*β* (g mg^−1^)	0.32
	*R* ^2^	0.902
	*χ* ^2^	32.1
Intra-particle diffusion	*K*_id_ (mg g^−1^ min^−1/2^)	1.96
	*C*_i_ (mg g^−1^)	1.0 × 10^−8^
	*R* ^2^	0.667
	*χ* ^2^	106.87

The Elovich model, which is commonly associated with adsorption onto energetically heterogeneous surfaces and a progressive decrease in adsorption rate as the surface coverage increases, produced an *R*^2^ value of 0.902. The Elovich curve captures the general increase in adsorption capacity with time but shows a greater deviation from the experimental data than the pseudo-first-order model. Nevertheless, its relatively good correlation supports the presence of heterogeneous adsorption sites and a gradual decrease in the adsorption rate as these sites become occupied. The different kinetic-model fittings collectively indicate that the adsorption process should not be interpreted as a single elementary adsorption event. Rather, the rapid initial uptake followed by a slower approach to equilibrium suggests a multistage adsorption process, involving rapid external surface adsorption followed by slower diffusion and occupation of less accessible adsorption sites within the porous structure.

#### 3.5.7 Intraparticle-diffusion analysis

To further investigate whether diffusion within the pores of Geo-BT5/H represents the sole rate-controlling step, the experimental kinetic data were analyzed using the Weber–Morris intraparticle-diffusion model, as shown in Fig. 15D. The plot of *Q*_t_*versus t*^1/2^ does not pass through the origin and exhibits a pronounced deviation from a single straight-line relationship. The relatively low correlation coefficient obtained for the intraparticle-diffusion model (*R*^2^ = 0.667) further confirms that intraparticle diffusion alone cannot adequately describe the overall adsorption kinetics. The nonlinearity observed in Fig. 15D can be associated with the occurrence of multiple adsorption stages. The initial portion corresponds mainly to boundary-layer diffusion and rapid external-surface adsorption, during which IC molecules migrate from the bulk solution toward the external surface of Geo-BT5/H. The subsequent region reflects intraparticle diffusion, whereby IC molecules penetrate the mesoporous network and interact with adsorption sites located within the pore structure. The final region approaches a plateau as the available adsorption sites become increasingly occupied and the concentration gradient decreases. The absence of a line passing through the origin is particularly important because it demonstrates that intraparticle diffusion is not the sole rate-controlling mechanism.^105,106^ Instead, the overall adsorption rate is governed by a combination of external mass transfer, surface adsorption, and diffusion within the mesoporous structure. This interpretation is consistent with the developed textural characteristics of Geo-BT5/H, which possesses a Type IV/H3 mesoporous structure and an interconnected pore network capable of facilitating IC transport toward internal adsorption sites. The kinetic results therefore indicate that rapid initial adsorption followed by progressively slower pore diffusion and site occupation governs IC uptake by Geo-BT5/H. The relatively good agreement of the pseudo-first-order model with the experimental data, together with the poor correlation obtained from the single intraparticle-diffusion model, confirms that the adsorption process involves multiple consecutive transport and adsorption steps rather than being controlled exclusively by intraparticle diffusion.

#### 3.5.8 Interference study

The influence of coexisting species on the adsorption of indigo carmine (IC) onto the Geo-BT5/H adsorbent was investigated to evaluate the selectivity and practical applicability of the synthesized adsorbent in multicomponent systems. As shown in Fig. 16, the experiments were performed using 50 mL of solution containing 20 mg L^−1^ IC at pH 2.0, with an adsorbent dose of 0.4 g, a contact time of 120 min, and a temperature of 25 ± 1 °C. Methylene blue (MB), Congo red (CR), neutral red (NR), and Cu^2+^ ions were selected as representative interfering species. These species provide different types of potential competition because MB and NR are cationic dyes, CR is an anionic dye, and Cu^2+^ represents an inorganic cation. In the absence of interfering species, Geo-BT5/H exhibited the highest removal efficiency for IC, reaching approximately 90%. The introduction of interfering species caused a moderate decrease in IC removal, although the adsorbent retained substantial adsorption capability in all investigated systems. Overall, the approximate trend observed in Fig. 16 can be expressed as: IC alone (∼90%) > IC + MB (∼82%) > IC + NR (∼78%) > IC + Cu^2+^ (∼75%) > IC + CR (∼72%) > IC + Cu^2+^ + CR (∼70%). This trend clearly confirms that coexisting species influence IC adsorption to different degrees. More importantly, Geo-BT5/H maintained relatively high IC removal efficiencies of approximately 70–82% in all tested interference systems, even though the single-component removal efficiency was approximately 90%. The relatively limited reduction observed in the presence of chemically different interfering species indicates that Geo-BT5/H possesses good selectivity and substantial tolerance toward matrix interference. The adsorbent can therefore efficiently retain its ability to remove IC in the presence of competing organic dyes and metal ions. These results are particularly encouraging from an application perspective because contaminated waters commonly contain mixtures of dyes, dissolved ions, and other chemical species rather than a single isolated pollutant. Accordingly, the interference study demonstrates the potential robustness of Geo-BT5/H for selective IC removal from complex aqueous matrices, while further investigation using actual wastewater samples would be valuable for confirming its performance under realistic operating conditions.

**Fig. 16:**
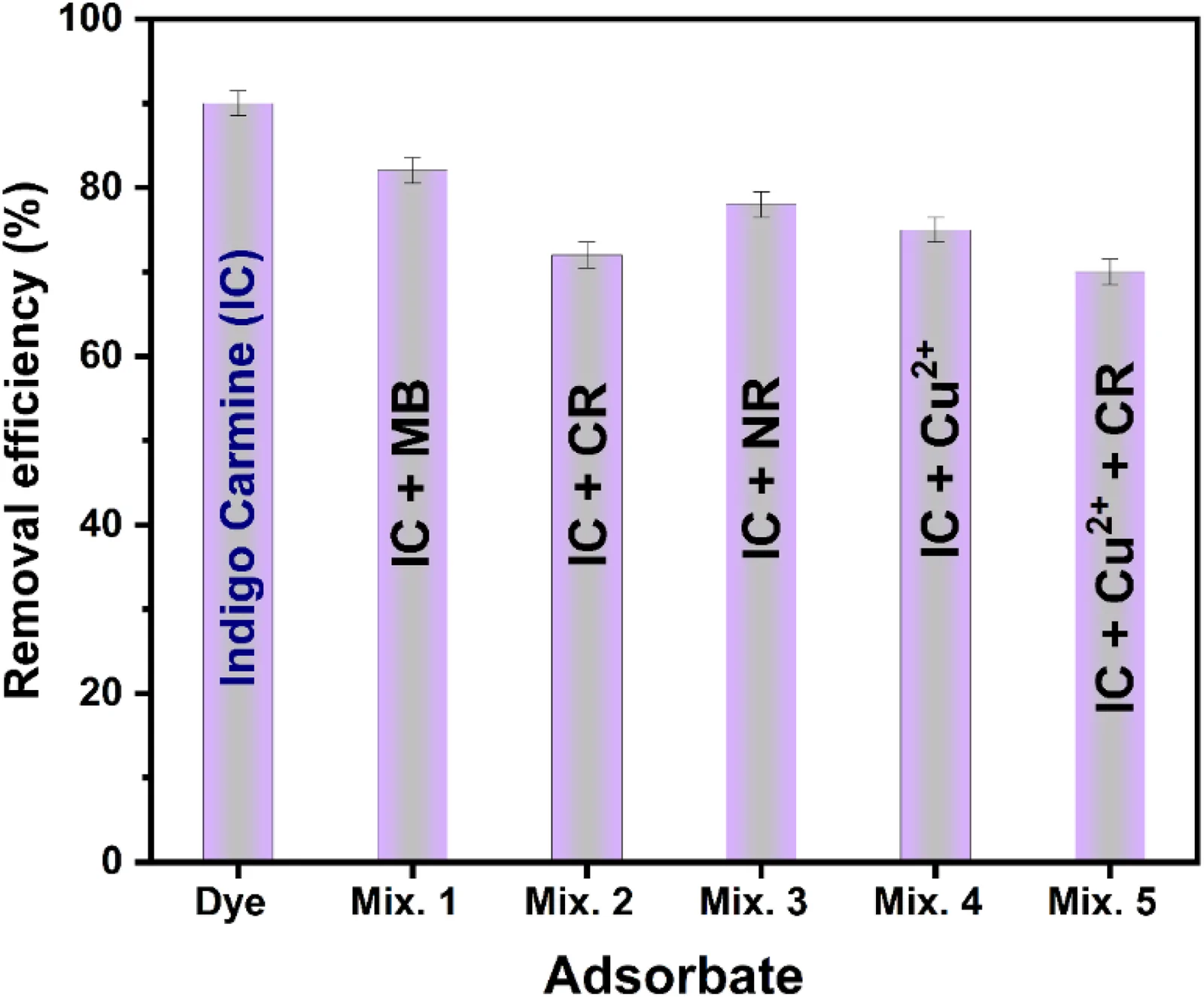
Impact of interfering species on the adsorption of indigo carmine (IC) onto Geo-BT5/H adsorbent under the following experimental conditions: solution volume = 50 mL, adsorbent dose = 0.4 g, pH = 2.0, contact time = 120 min, temperature = 25 ± 1 °C, and initial IC concentration = 20 mg L^−1^. MB: methylene blue; CR: Congo red; NR: neutral red; and Cu^2+^: copper ions.

#### 3.5.9 Reusability of adsorbent

The practical applicability of an adsorbent largely depends on its ability to maintain adsorption performance after repeated use. Fig. 17 demonstrates that Geo-BT5/H preserves a high dye removal efficiency throughout successive adsorption–desorption cycles, with only a slight gradual decrease after repeated regeneration. The slight decline in adsorption efficiency may be attributed to partial occupation of adsorption sites by strongly retained dye molecules, incomplete regeneration of the geopolymer surface, or minor blockage of pore channels after repeated adsorption cycles.^107^ Nevertheless, the decrease remains limited, indicating excellent structural stability of the developed adsorbent.^106^ The structural integrity of Geo-BT5/H after repeated adsorption is further confirmed by the FTIR and XRD patterns shown in Fig. 18. The characteristic FTIR bands corresponding to Si–O–Si, Si–O–Al, hydroxyl groups, and geopolymeric frameworks remain essentially unchanged before and after adsorption, indicating preservation of the principal functional groups responsible for dye uptake. Likewise, the XRD patterns exhibit negligible changes in the crystalline structure, confirming that the zeolitic and aluminosilicate frameworks remain intact during repeated adsorption–desorption operations. The combined adsorption and characterization results demonstrate that hydrothermally synthesized Geo-BT5/H possesses excellent regeneration capability together with high structural stability. These characteristics, coupled with its low production cost, high mechanical strength, and developed mesoporous structure, make this material a promising sustainable adsorbent for repeated treatment of dye-containing industrial wastewater.

**Fig. 17:**
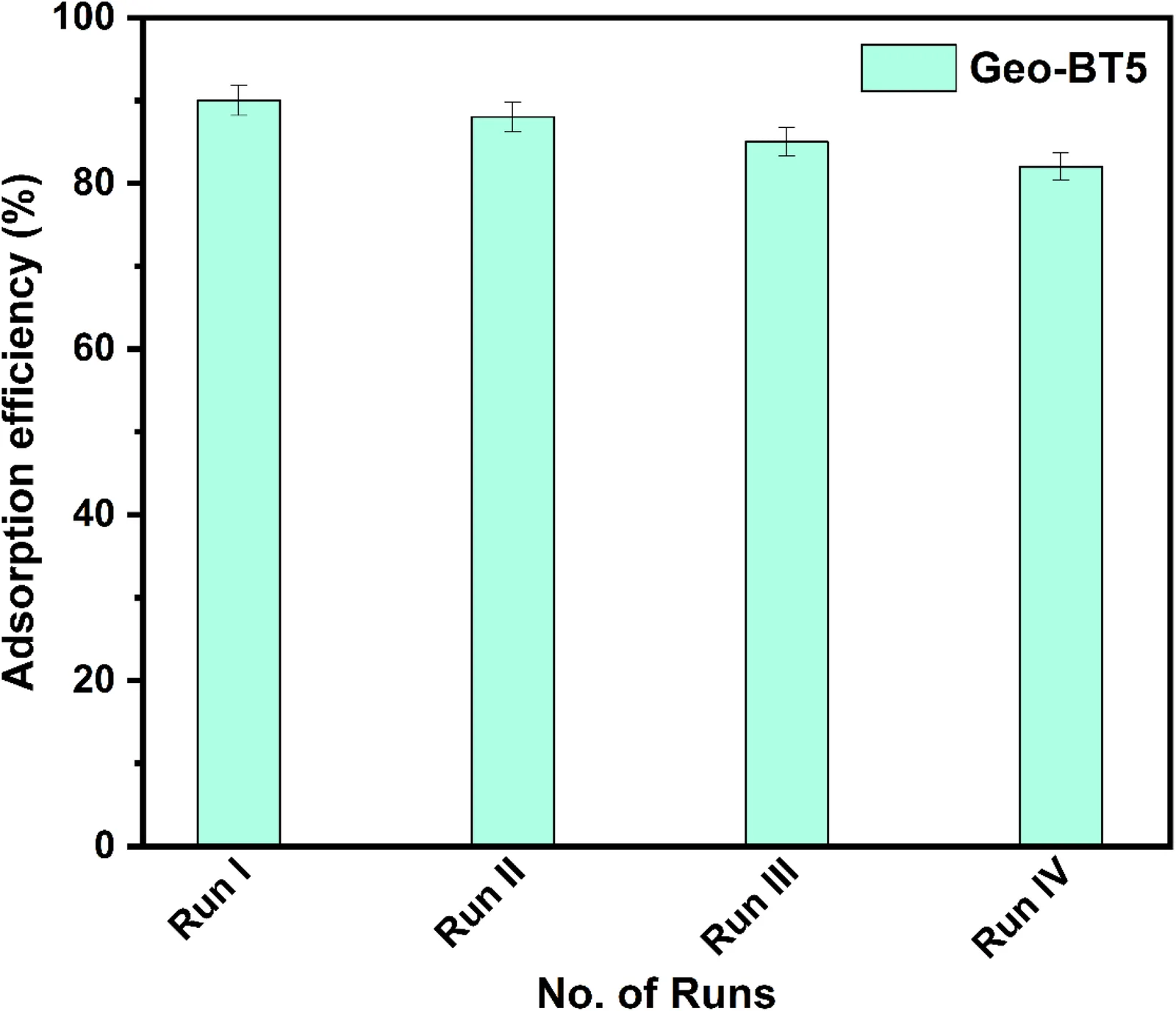
The recyclability of Geo-BT5/H operated on IC dye (contact time = 120 minutes, temperature = 293 K, volume = 50 mL, adsorbent dose = 0.4 g, pH ≈ 2).

**Fig. 18:**
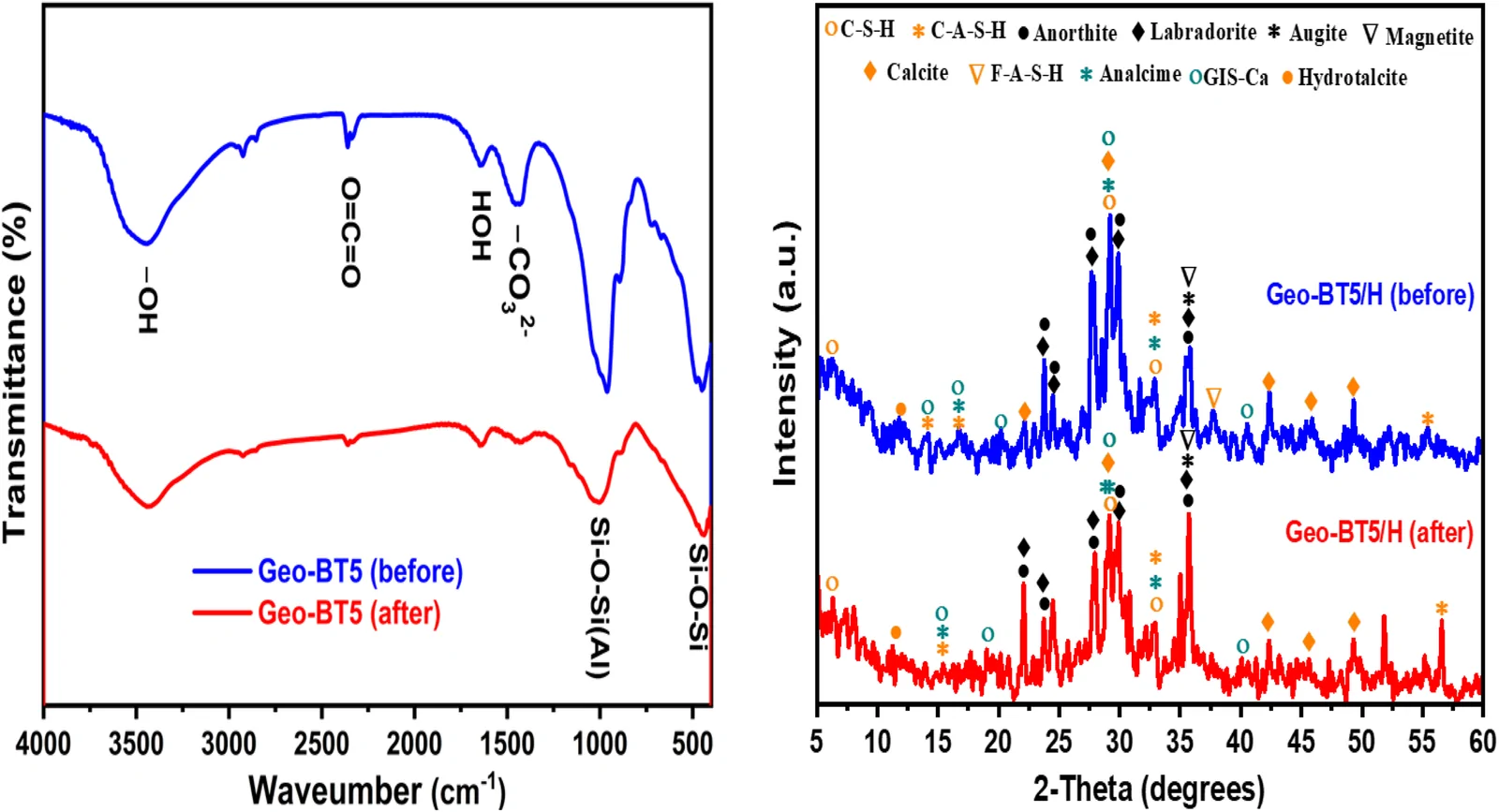
FTIR spectra (left) and XRD patterns (right) of the recycled adsorbent before and after the adsorption experiments.

#### 3.5.10 Comparison with other adsorbents

To evaluate the adsorption performance of the developed Geo-BT5/H adsorbent toward indigo carmine (IC), its adsorption capacity was compared with those of various adsorbents previously reported for IC removal, as summarized in Table 8. Such a comparison is important for assessing the relative effectiveness and potential applicability of the prepared material. In particular, the maximum adsorption capacity (*q*_max_) provides a useful quantitative indicator of the amount of IC that can be accommodated per unit mass of adsorbent. Nevertheless, direct comparison of adsorption capacities should be made cautiously because the reported values are generally obtained under different experimental conditions, including solution pH, temperature, initial dye concentration, adsorbent dosage, contact time, ionic strength, and equilibrium-model assumptions. For indigo carmine specifically, the reported adsorption capacities include 11–12.6 mg g^−1^ for an activated carbon/volcanic ash composite, 3.5–4.2 mg g^−1^ for a geopolymer prepared from biomass ash, 2.5–5 mg g^−1^ for a graphene oxide/poly(acrylamide)/formic acid system, and 4.9 mg g^−1^ for a previously reported slag-based geopolymer. The present basalt-based geopolymer exhibited a maximum adsorption capacity of approximately 6 mg g^−1^. The comparison indicates that the adsorption capacity of Geo-BT5/H is within the range reported for geopolymer-based and other low-cost adsorbents for indigo carmine. Although certain highly engineered adsorbents exhibit higher capacities, the present material should be considered in terms of its multifunctionality, low-cost raw materials, mechanical strength, developed mesoporosity, and potential reusability, rather than adsorption capacity alone. In particular, the use of basalt and slag as abundant mineral resources provides an attractive route for producing a mechanically robust adsorbent without relying on expensive carbonaceous nanomaterials or highly engineered synthetic adsorbents. So, the newly added discussion therefore provides an appropriate literature benchmark and clarifies the relative adsorption performance of Geo-BT5/H.

**Table 8:** Comparison of adsorption capacity onto the developed Geo-BT5/H with other reported adsorbents

Adsorbent	Dye	*q*_max_ (mg g^−1^)	Reference
Hydroxyapatite nanoparticles	Methylene blue	40	108
Acid treated kaolin	Methylene blue	64.5	109
Alkali-activated blast furnace slag	Methylene blue	39.5	110
Ca/Al layered hydroxide-biochar	Methylene blue	32.5	111
Metakaolin-based geopolymer	Methylene blue	7.6	112
Alkali-activated geopolymer	Methylene blue	210	71
Xylan-gelatin cross-linked hydrogel	Methylene blue	35.5	113
Activated carbon/volcanic ash composite	Indigo carmine	11, 12.6	114
Geopolymer from biomass ash	Indigo carmine	3.5, 4.2	115
Graphene oxide/poly (acrylic amide/formic acid)	Indigo carmine	2.5, 5	116
Slag-based geopolymer	Indigo carmine	4.9	36
Basalt-based geopolymer	Indigo carmine	6	This work

### 3.6 Cost analysis and environmental impact

A preliminary CO_2_ emissions and cost assessment was performed to evaluate the potential environmental and economic benefits of the developed high basalt geopolymer relative to ordinary Portland cement (OPC). Three systems were analyzed: OPC, the normally cured geopolymer (Geo-BT5/N), and the hydrothermally cured one (Geo-BT5/H) at 150 °C/12 h. The calculations were based on 1 ton of precursor/binder. For the geopolymer mixtures, the precursor consisted of 50 wt% GGBFS and 50 wt% basalt, while NaOH was added at 5 wt% relative to the precursor mass. The adopted CO_2_-emission factors were 944, 26.5, 7.65 and 1232 kg CO_2_/t for OPC, GGBFS, basalt and NaOH, respectively.^64,66,80,117,118^ The electricity emission factor was taken as 0.55 kg CO_2_/kWh.^60,119^ For the cost analysis, the corresponding prices were 90, 46, 3.84, and 408 $ per t, respectively, while electricity was valued at 0.044 $ per kWh.^58,80^

As represented in Table 9, the calculated raw-material CO_2_ emission of Geo-BT5/N was approximately 78.68 kg CO_2_/t, compared with 944 kg CO_2_/t for OPC, corresponding to an estimated reduction of about 91.7%. This significant reduction is primarily due to substituting OPC with GGBFS and, especially, the high proportion of natural basalt, which has relatively low embodied carbon. For Geo-BT5/H, the additional energy associated with hydrothermal curing was also considered. At laboratory scale, the specimens were hydrothermally treated in small-sealed autoclave reactors placed inside an oven. For the preliminary scale-up assessment, an industrial curing scenario was considered using an oven with a heating power of 200 kW and an assumed batch capacity of 3 tons; these specifications were selected based on several industrial ovens. The selected batch capacity was accounted the space occupied by the autoclave vessels and racks and was based on the dimensional ranges reported for industrial drying ovens. Hydrothermal curing was considered at 150 °C/12 h. Although under practical industrial operation, the heating system would normally cycle (on/off) after the target temperature is reached rather than operate continuously at full power for the entire 12 h, the oven was assumed to operate continuously to obtain an upper-bound estimate. Under this assumption, the total electricity consumption was estimated at approximately 2400 kWh per batch (3 tons), corresponding to 800 kWh t^−1^ of processed material. Based on these calculations, the curing stage contributed approximately 440 kg CO_2_/t, increasing the total estimated CO_2_ emission of Geo-BT5/H to about 518.68 kg CO_2_/t. Despite this additional energy demand, the hydrothermally cured geopolymer still exhibited an estimated 45.1% reduction in CO_2_ emissions relative to OPC.

**Table 9:** CO_2_ emission and cost analysis for preparing 1 ton of OPC and GGBFS/basalt-geopolymeric composites

Composite	Ingredient	CO_2_-emission from each item (kg)	Total CO_2_-emission (kg)	Cost for each item ($)	Total cost ($)
OPC	1000 kg OPC	944	944	90	90
Geo-BT5/N	500 kg GGBFS	13.25	78.67	23	45.32
	500 kg basalt	3.825		1.92	
	50 Kg NaOH	61.6		20.4	
Geo-BT5/H	500 kg GGBFS	13.25	518.67	23	80.52
	500 kg basalt	3.825		1.92	
	50 Kg NaOH	61.6		20.4	
	Electricity	440		35.2	

The preliminary cost analysis showed a similar tendency. The estimated raw-material cost of Geo-BT5/N was approximately 45.32 $ per t, compared with 90 $ per t for OPC, corresponding to a cost reduction of about 49.6%. High basalt incorporation contributed significantly to this reduction because basalt is a relatively low-cost, locally abundant natural resource. For Geo-BT5/H, the conservative curing-energy assumption resulted in an additional electricity cost of approximately 35.20 $ per t. Consequently, the total estimated cost increased to approximately 80.52 $ per t. Despite the additional hydrothermal-curing cost, this value remained about 10.5% lower than that of OPC. Finally, it can be concluded that incorporating 50 wt% basalt into the slag-based geopolymer can provide substantial economic and environmental benefits.

## 4 Conclusion

A sustainable low-carbon alkali-activated geopolymer was successfully developed using equal proportions of ground granulated blast-furnace slag (GGBFS) and Egyptian natural basalt (BT). The effects of alkali concentration and curing regime on the physicochemical, mechanical, and microstructural properties of the geopolymer were systematically evaluated. The incorporation of BT into the slag-based geopolymer increased setting times while maintaining compliance with ASTM limits and significantly improved workability compared with OPC. Furthermore, increasing the NaOH concentration from 5 to 10 wt% further delayed setting and enhanced flowability. Hydrothermal curing markedly enhanced geopolymerization compared with ambient curing, producing a denser and more refined microstructure with crystalline C-A-S-H and zeolitic phases, while the Geo-BT5/H composite achieved the highest compressive strength of 53.3 MPa after 12 h of autoclave treatment.

Acid activation increased the specific surface area of Geo-BT5/H powder by 28.43%, enhancing its mesoporosity and adsorption potential. The Indigo Carmine (IC) adsorption capacity increased with increasing initial dye concentration and temperature. Thermodynamic analysis confirmed that IC adsorption was spontaneous, endothermic, and entropy-driven, with enhanced adsorption occurring at elevated temperatures. Equilibrium and kinetic investigations demonstrated that IC adsorption onto Geo-BT5/H was adequately described by the Redlich–Peterson model and proceeded through multiple stages, involving rapid surface uptake followed by diffusion toward less accessible adsorption sites. The results also indicated that intraparticle diffusion was not the sole rate-controlling mechanism. Finally, the hydrothermally cured slag-basalt geopolymer demonstrated dual functionality by combining high mechanical strength with efficient and reusable dye adsorption, making it a promising sustainable material for construction and wastewater treatment applications.

Future studies should evaluate the long-term durability of the developed binders through sulfate, chloride, and acid resistance tests; freeze-thaw cycling; fire resistance; high-dose radiation resistance; water absorption; leaching assessments; and microbial deterioration. These investigations will provide a more comprehensive understanding of the long-term performance and practical applicability of the developed material.

## Conflicts of interest

The authors declare that they have no known competing financial interests or personal relationships that could have appeared to influence the work reported in this paper.

## Data Availability

The data supporting the findings of this study are available from the corresponding author upon reasonable request.
